# Mito-Tempol and Dexrazoxane Exhibit Cardioprotective and Chemotherapeutic Effects through Specific Protein Oxidation and Autophagy in a Syngeneic Breast Tumor Preclinical Model

**DOI:** 10.1371/journal.pone.0070575

**Published:** 2013-08-05

**Authors:** Jennifer S. Dickey, Yanira Gonzalez, Baikuntha Aryal, Steven Mog, Asako J. Nakamura, Christophe E. Redon, Ulrich Baxa, Elliot Rosen, Gang Cheng, Jacek Zielonka, Palak Parekh, Karen P. Mason, Joy Joseph, Balaraman Kalyanaraman, William Bonner, Eugene Herman, Emily Shacter, V. Ashutosh Rao

**Affiliations:** 1 Division of Therapeutic Proteins, Center for Drug Evaluation and Research, Food and Drug Administration, Bethesda, Maryland, United States of America; 2 Center for Food Safety and Applied Nutrition, Food and Drug Administration, College Park, Maryland, United States of America; 3 Laboratory of Molecular Pharmacology, National Cancer Institute, Bethesda, Maryland, United States of America; 4 Electron Microscopy Laboratory, Advanced Technology Program, Frederick National Laboratory for Cancer Research, Frederick, Maryland, United States of America; 5 Department of Biophysics and Free Radical Research Center, Medical College of Wisconsin, Milwaukee, Wisconsin, United States of America; 6 Division of Drug Safety Research, Center for Drug Evaluation and Research, Food and Drug Administration, Silver Spring, Maryland, United States of America; Duke University Medical Center, United States of America

## Abstract

Several front-line chemotherapeutics cause mitochondria-derived, oxidative stress-mediated cardiotoxicity. Iron chelators and other antioxidants have not completely succeeded in mitigating this effect. One hindrance to the development of cardioprotectants is the lack of physiologically-relevant animal models to simultaneously study antitumor activity and cardioprotection. Therefore, we optimized a syngeneic rat model and examined the mechanisms by which oxidative stress affects outcome. Immune-competent spontaneously hypertensive rats (SHRs) were implanted with passaged, SHR-derived, breast tumor cell line, SST-2. Tumor growth and cytokine responses (IL-1A, MCP-1, TNF-α) were observed for two weeks post-implantation. To demonstrate the utility of the SHR/SST-2 model for monitoring both anticancer efficacy and cardiotoxicity, we tested cardiotoxic doxorubicin alone and in combination with an established cardioprotectant, dexrazoxane, or a nitroxide conjugated to a triphenylphosphonium cation, Mito-Tempol (4) [Mito-T (4)]. As predicted, tumor reduction and cardiomyopathy were demonstrated by doxorubicin. We confirmed mitochondrial accumulation of Mito-T (4) in tumor and cardiac tissue. Dexrazoxane and Mito-T (4) ameliorated doxorubicin-induced cardiomyopathy without altering the antitumor activity. Both agents increased the pro-survival autophagy marker LC3-II and decreased the apoptosis marker caspase-3 in the heart, independently and in combination with doxorubicin. Histopathology and transmission electron microscopy demonstrated apoptosis, autophagy, and necrosis corresponding to cytotoxicity in the tumor and cardioprotection in the heart. Changes in serum levels of 8-oxo-dG-modified DNA and total protein carbonylation corresponded to cardioprotective activity. Finally, 2D-electrophoresis/mass spectrometry identified specific serum proteins oxidized under cardiotoxic conditions. Our results demonstrate the utility of the SHR/SST-2 model and the potential of mitochondrially-directed agents to mitigate oxidative stress-induced cardiotoxicity. Our findings also emphasize the novel role of specific protein oxidation markers and autophagic mechanisms for cardioprotection.

## Introduction

Many of the most commonly used anticancer agents induce cardiac toxicity as a dose-limiting side effect [1]. Anticancer therapies that are known to cause cardiac side effects originate from a wide array of drug classes, from anthracyclines such as the topoisomerase II poison doxorubicin to protein-based drugs such as interleukin-2 and trastuzumab [2], [3]. Because there is no well-defined predictor of whether an anticancer agent will induce cardiac toxicity while reducing tumor burden, often these deleterious side effects are only discovered late in the drug development process or after years of use in the clinic [4]. Doxorubicin increases reactive oxygen species (ROS) levels in the mitochondria through selective sequestering, redox cycling, and an iron-mediated mechanism. ROS-induced protein carbonylation is one of the most physiologically-relevant oxidative modifications of proteins because it marks affected proteins for proteosomal degradation [5]. The protein damage caused by oxidative stress is directly correlated with the increased number of carbonyl groups in proteins [6]. Since cardiac cells have lower levels of antioxidant defenses including superoxide dismutase (SOD) and catalase, the heart is extremely sensitive to ROS [7]. Dexrazoxane is an iron chelator that prevents oxidative stress and helps to mitigate the cardiotoxic effects of doxorubicin [8]. Dexrazoxane does not appear to affect the anticancer ability of doxorubicin in the clinic [9].

One limitation in our ability to evaluate cardioprotective anticancer agents is the lack of physiologically-relevant and immune-proficient animal models that can simultaneously address anticancer efficacy and monitor changes in adverse cardiac effects. Typically, animal studies have relied on two different systems for independently assaying anti-tumor and cardioprotective potential. For example, the xenografted nude mouse is typically used to determine antitumor activity while the spontaneously hypertensive rat (SHR) is the common model for cardiotoxicity studies [10]. The SHR model has demonstrated good correlation between cardiomyopathy induced by anthracyclines and increases in serum levels of cardiac troponin-T, a standard biomarker of cardiotoxicity [11], [12]. While the Fisher and Wistar rat models have been applied to the study of tumor reduction and cardiotoxicity previously, the SHR model is uniquely suited for cardiac studies because of the low inter-individual variation, uniform polygenic disposition, and well-characterized biochemical responses to anthracycline toxicity [11], [13]–[15]. The SHR is also considered advantageous because the highly reproducible cardiac lesions and organ damage that develop in this animal in response to anthracyclines are similar in both degree and type to humans [14], [15]. Finally, SHRs are suitably sized to obtain enough serum to allow low-abundance biomarker analysis [16]. However, the SHR model had not been fully developed for its ability to study both tumor reduction and cardiotoxicity. As such, the SHR model was instrumental in supporting the use of dexrazoxane as a cardioprotectant in humans [17].

Because it is critical to understand the interplay between cardiac health, the immune system, and tumor response in order to develop cardioprotectants, we optimized the immune-competent SHR model to assess both the cardiac safety and anticancer efficacy of drugs. The immune system, particularly in the tumor microenvironment, plays a crucial role in modulating tumor progression and response to therapy [18]. The syngeneic breast tumor cell line SST-2 has been previously used for studying anticancer immune responses and metastasis but not applied to the study of cardiotoxicity mechanisms or the development of cardioprotective and chemotherapeutic strategies [19]–[22]. Because the SHR/SST-2 model has a competent immune system and displays successful tumor uptake, we hypothesized that it would allow physiologically-relevant monitoring of cardiac toxicity in addition to antitumor activity. We further hypothesized that cytotoxic mechanisms in the heart and tumor are likely a combination of apoptosis, autophagy, and necrosis. As autophagy is a known survival mechanism, we tested whether tissues exhibit levels of autophagy or apoptosis that are reflective of the cytoprotective or cytotoxic outcome [23], [24]. In order to validate our animal model, we used doxorubicin as a positive control due to its well-known anticancer and cardiotoxic effects.

Dexrazoxane is currently used in combination with doxorubicin for cardioprotection [25]. However, there is a need for better cardioprotectants because dexrazoxane does not eliminate the potential for cardiomyopathy by anthracyclines and newer anticancer agents that cause cardiotoxicity, such as trastuzumab, interleukin-2, and tyrosine kinase inhibitors, which are not known to elicit cardiotoxicity through iron-mediated oxidative stress [26]–[28]. In addition to testing dexrazoxane and the autophagic response in the SHR/SST-2 model, the novel therapeutic mitochondrialy-targeted Tempol (Mito-T) was also investigated. Tempol is a well-known SOD mimetic that has been examined previously as an antioxidant and radio-protectant [29]. Tempol has also been shown to alleviate oxidative stress and cardiac toxicity induced by doxorubicin in rats [30]. Mito-T (4) consists of the tempol moiety bound to a triphenylphosphonium cation that serves to drive the molecule into the mitochondria. Mito-T (4) also has the advantage of being capable of rapid uptake and recycling by the mitochondrial respiratory chain [31]. Thus, Mito-T (4) might be especially efficacious in mitochondria-rich organs such as the heart, which are considered the source of damaging excess ROS by doxorubicin [32].

Results of our study indicate that the dual-purpose SHR/SST-2 experimental system is able to accurately reflect both the antitumor activity and cardiotoxicity of therapeutics. In addition to novel findings of cardioprotection by Mito-T (4), we identify the mechanisms by which autophagy and protein oxidation might impact cardiac cell survival.

## Materials and Methods

### Chemicals

The compounds dexrazoxane and doxorubicin were purchased from Pharmacia Laboratories (Columbus, OH). Mito-T (4) was synthesized as described below.

### Synthesis of Mito-T (4)

Mito-T (4) synthesis involves three steps as shown in the scheme provided in Figure S1. In the first step, 1.72 g of Tempol was refluxed in benzene (100 ml) with 0.5 g of sodium hydride (60% suspension in paraffin oil) under a nitrogen atmosphere for 24 h. In the second step, the reaction mixture was cooled in an ice bath and 6 g of 1,4-dibromobutane was added to the mixture and further refluxed for 24 h. The reaction mixture was again cooled and 10 ml of water was added carefully to neutralize the excess sodium hydride. After adding 50 ml of ether, the mixture was shaken in a separatory funnel with 50 ml water. The organic layer was collected and solvent removed to obtain a red oil. This oil was purified on a column of silica gel 60 and first eluted with hexane to remove the excess of dibromobutane. The desired product bromobutylether of Tempol was eluted with a mixture of ether and hexane (1∶1) as a slow moving orange band. The homogeneous fractions were collected and solvent removed to get the pure product as a red oil (2.0 g). In the third step, the bromobutylether of Tempol (2 g) and triphenylphosphine (2 g) were taken in a flask and 10 ml of dioxane was added and heated under a nitrogen atmosphere for 48 h in an oil bath kept at 90°C. The solvent was removed by blowing nitrogen gas and stirred with 50 ml of dry ether to get a precipitate of Mito-T (4) as a semisolid. The ether was decanted off and the residue was dissolved in 5 ml of dichloromethane to obtain a red solution. This solution was added drop-wise to ether (50 ml) while stirring and the precipitated product was separated by decantation. This process of precipitation from ether was repeated for a total of four times to obtain a pure Mito-T (4) (3.0 g) as a brown hygroscopic powder. The purity of the product was ascertained by performing HPLC and LC/MS (mass  = 489.282, Figure S1).

### Cell culture

Rat breast cancer cells (SST-2) were obtained from Dr. Nozomu Koyangi (Eisai Co., Ltd. Clinical Research Center, Tokyo, Japan) and maintained in RPMI medium containing 5% heat-inactivated fetal bovine serum (FBS), 2 mM L-Glutamine, 1 mM sodium pyruvate, and 50 μM β-mercaptoethanol at 37°C in 5% CO_2_ [33], [34]. The SST-2 cell line was last authenticated in August 2011 by RADIL at the University of Missouri (now IDEXX RADIL, Columbia, MO). The mycoplasma- and viral contaminant-free status was also confirmed at the same time by RADIL.

### Animals

Female SHRs (10 weeks of age) were obtained from Harlan Laboratories, Inc. (Indianapolis, IN) and were housed individually in an environmentally controlled room (18–21°C, 40–70% relative humidity, 12-h light/dark cycle). The study commenced after a 7-day acclimation period. Rats were fed Certified Purina Rodent Chow #5002 (Ralston Purina Co., St. Louis, MO) and water *ad libitum*. The experimental protocol was approved by the Institutional Animal Care and Use Committee, Center for Drug Evaluation and Research, FDA, and conducted in an AAALAC-accredited facility. All procedures for animal care and housing were in compliance with the Guide for the Care and Use of Laboratory Animals, 1996 (Institute of Laboratory Animal Resources).

### Animal study design

SHRs were subcutaneously implanted with exponentially-growing SST-2 cells in the right mammary fat pad. The following day, nine groups of 10 SHR each were treated with saline, doxorubicin (10 mg/kg), Mito-T (4) (5 mg/kg or 25 mg/kg) or dexrazoxane (50 mg/kg), alone or in combination with doxorubicin, or received no treatment. Doxorubicin was administered intravenously (IV) via the lateral tail vein. Dexrazoxane and Mito-T (4) were given by intraperitoneal injection (IP) either alone or in combination. The animals were observed for 14 days following treatment during which they were weighed at days 7, 10, and 14. After two weeks SHRs were euthanized under isoflurane anesthesia. The heart was weighed and heart and tumor tissues were frozen or fixed in 10% formalin solution for electron microscopy. Two deaths were recorded in the 25 mg/kg Mito-T (4) and doxorubicin combination treatment group.

### Clinical chemistry and hematology analysis

At necropsy, blood samples were collected from the inferior vena cava into blood collection tubes and centrifuged to obtain serum samples. Clinical chemistry determinations were performed using the VetScan Analyzer (Abaxis, Inc., Union City, CA). An aliquot of whole blood was also collected in order to assess various hematological parameters (Abaxis, Inc.).

### Histopathological studies

Portions of the hearts were embedded in glycol methacrylate plastic resin, sectioned (1 μm) and stained with toluidine blue. Other portions of the heart were embedded in paraffin, cut into 5 μm sections, and stained with hematoxylin and eosin. Histologically, cardiac sections were scored (0 to 3) for cardiomyopathy (cardiomyocyte cytoplasmic vacuolization and myofibrillar loss) by a board-certified veterinary pathologist using a previously reported scoring system [12], [35]. A ranked score approach of the ordinal data obtained from the lesion scores was used. For the histology scoring, a non-parametric test (Mann-Whitney U test) was applied. Using the raw data from each animal group (n = 5), the significant groups are ranked 1–4 when compared to saline. The two groups ranked 5–6 are not significantly different from the saline control (p<0.05).

### Analysis of Mito-T (4) in mitochondrial fractions from cells and tissues

#### Sample preparation

Tissue samples (heart or tumor) were placed in bead homogenizer tubes (100–200 mg of wet tissue per tube) with 3 ceramic beads (2.8 mm) and 0.5 ml of homogenization medium (220 mM mannitol, 70 mM sucrose, 10 mM HEPES, and 2 mM EDTA at pH 7.4). Samples were homogenized with Bead Ruptor 24 homogenizer (Omni International, Kennesaw, GA) and transferred to 1.5 mL centrifuge tubes. Cells were lysed in the same homogenization medium by drawing cell suspension 20 times through an 28G1/2” insulin syringe. Tissue homogenates and cell lysates were centrifuged for 10 min at 600 *g* and 4°C to remove cell/tissue debris and nuclear fraction. Supernatants were collected and centrifuged again for 10 min at 15,000 *g* and 4°C to isolate mitochondria. The supernatant was removed and mitochondrial pellet was resuspended in 200 µl of the homogenization medium and sonicated for 1 min. The mitochondrial homogenate was taken for protein assay and the remainder was used for extraction of Mito-T (4) in a 2∶1 dichloromethane-methanol mixture. The extraction was repeated, organic layers combined, and the solvent was removed with SpeedVac (Thermo Fisher Scientific Inc., Waltham, MA). Immediately before LC-MS analysis the dry residue was dissolved in 100 µl of a 1∶1 water-methanol mixture spiked with n-butyltriphenylphosphonium bromide as an internal standard. The solution was vortexed for 10 min and centrifuged for 30 min at 20,000 *g* and 4°C. The supernatant was transferred into HPLC vials and analyzed by HPLC-MS/MS.

#### LC-MS/MS analysis

Mito-T (4) was analyzed using the Nexera UHPLC system equipped with a UV-Vis absorption detector and LC-8030 triple quadrupole mass detectors (Shimadzu, Columbia, MD). Samples were injected into the Kinetex Phenyl-Hexyl column (Phenomenex, Torrence,CA), which was pre-equilibrated with a 9∶1 water-acetonitrile mixture containing 0.1% formic acid. Analytes were eluted by an increase in the fraction of acetonitrile from 10 to 60% over 4 min. Mito-T (4) was detected by monitoring an MRM transition 489.0 → 474.2 and eluted at 2.82 min and n-butyltriphenylphosphonium was detected using a transition 319.10 → 183.0 (retention time of 2.56 min).

### Immunocytochemical analysis

Tissues were touch-printed onto slides, fixed with 4% paraformaldehyde, and permeabilized with 70% ethanol as described previously [36]. Slides were stained with the rabbit anti-γ-H2AX (1∶500) (Novus, Littleton, CO) and Alexa-Fluor 488 anti-rabbit secondary antibody (Molecular Probes, Eugene, OR). Cells were visualized with green, DAPI, or phase contrast filters using a 40× objective on a Zeiss LSM510 laser scanning confocal microscope (Carl Zeiss Microimaging, Thornwood, NY). Image analysis was performed using the Adobe Photoshop suite (Adobe Systems Inc, San Jose, CA). Active caspase-3 staining of paraffin-embedded tissue sections was performed by Immunostain (Derwood, MD).

### Protein extraction from rat tissue

Approximately 100 mg of tissue was excised from each frozen rat heart. The tissues were washed with PBS containing protease inhibitors, phosphatase inhibitors, and 1 mM diethylene triaminepentaacetic acid (DTPA). The samples were then homogenized in RIPA buffer (25 mM TrisCl, pH7.6, 150 mM NaCl, 1% NP-40, 1% sodium deoxycholate and 0.1% SDS) containing protease inhibitors, phosphatase inhibitors, and 1 mM DTPA. Supernatants were collected and SDS was added to a final concentration of 3%. Protein quantification was performed using a BCA protein assay kit (Thermo Scientific, Rockford, IL).

### Western blotting

Total protein (30 μg) was loaded and resolved in a Novex 4–12% Bis-Tris gel in MES SDS running buffer (Life Technologies, Grand Island, NY). Protein was transferred to an Immobilon-P PVDF membrane (Millipore, Billerica, MA) and probed with either anti-LC3-II (Novus Biologicals, Littleton, CO) or anti-GAPDH (Imgenex, San Diego, CA) as a loading control.

### Caspase-3 fluorometric assay

A fluorometric immunosorbent enzyme assay kit was used to detect activity of caspase-3 as described by the manufacturer (Roche Applied Science, Indianapolis, IN). Briefly, whole cell lysates were used for capturing caspase-3 on an anti-caspase-3 coated microplate well. The captured protein was then incubated with caspase substrate Ac-DEVD-fmk. The increased fluorescence corresponded to free AFC dye, indicating enzymatic activity.

### Transmission electron microscopy

Animal tissue samples were fixed with 4% formaldehyde and 2% glutaraldehyde in 0.1 M cacodylate buffer, pH 7.2 (Tousimis, Rockville, MD) and trimmed to about 1.0–2.5 mm^3^ thickness. Tissue blocks were postfixed in 1% osmium tetroxide for 1 h and *en bloc* stained in 0.5% uranyl acetate for 1 h. The samples were then dehydrated in a graded series of 35, 50, 70, and 100% ethanol and exchanged for propylene oxide. The tissue samples were then infiltrated with 1∶1 propylene oxide and epoxy resin overnight, allowing for evaporation of propylene oxide, and finally embedded in 100% epoxy resin the next day. Polymerization of resin was cured for 3 d at 55°C. Thin sections of 70–90 nm were cut on an Leica UC6 ultramicrotome (Leica Microsystems, Buffalo Grove, IL), stained with uranyl acetate and lead citrate, lightly carbon coated, and imaged in a Hitachi 7650 transmission electron microscope (Hitachi High-Tech, Schaumburg, IL) operating at 80 kV. Images were taken with an AMT digital camera (Advanced Microscopy Techniques, Woburn, MA).

### 8-hydroxy-2′-deoxyguanosine (8-oxo-dG) ELISA

The concentration of 8-oxo-dG in serum samples was measured using an in vitro enzyme-linked immunosorbent assay (ELISA) kit method (Trevigen, Gaithersburg, MD). The serum samples were thawed and diluted five fold. An equal volume of monoclonal antibody and sample or standard (50 μL) was added in a 96-well microplate precoated with 8-oxo-dG. The plate was incubated at room temperature for 1 h and washed. HRP-conjugated secondary anti-mouse antibody (100 μL) was then added to each well and incubated for 1 h at room temperature. Upon removing the antibody, 100 μL of tetramethylbenzidine substrate was added to each well and incubated for 15 min at room temperature, before the reaction was stopped with the addition of 100 μL of an acid stop solution. Absorbance was measured with a spectrophotometer at 450 nm and the concentration of 8-oxo-dG in each sample was calculated by comparison to the standard curve.

### Determination of total protein carbonylation

Serum protein carbonylation was determined by using a modified procedure based on DNPH-derivatization as previously published [6], . Approximately 5 µg of total serum protein for each treatment sample was treated with 6% SDS in a 15 µL volume. An equal volume of 20 mM 2,4-dinitrophenyl hydrazine (DNPH) in 10% TFA was added and incubated at room temperature for 10 min. The reaction was neutralized with 14.5 µL of 2 M Tris in 30% glycerol and 7% 2-mercaptoethanol, and ran on two identical gels. One gel was used for coomassie staining for total protein quantification and another gel was used for Western blot analysis to determine the total protein carbonylation. After overnight transfer at 30 V, the membrane was blocked for one hour with blocking buffer (LI-COR, Lincoln, NE). The membrane was treated with goat anti-DNP primary antibody (Bethyl Laboratories Inc., Montgomery, TX) followed by donkey anti-goat IRDye 800CW secondary antibody (LI-COR, Lincoln, NE). DNP-derivatized carbonylated proteins were detected using the Odyssey infrared imaging system (LI-COR, Lincoln, NE). Quantitation of total protein and carbonylated protein was performed by densitometric analysis of the entire lane for gel staining and Western blot using Odyssey software. Densitometric values for protein carbonylation were normalized to total protein for the corresponding sample to calculate the relative protein carbonylation.

### Detection of carbonylated protein using 2D-gel electrophoresis

2D-gel electrophoresis for each serum sample was performed using the previously published protocol with minor modifications as described [38]. Serum samples were denatured in 6% SDS and treated with 20 mM DNPH in 10% TFA to a final concentration of 10 mM DNPH and incubated for 10 min at room temperature. Proteins were precipitated with trichloroacetic acid (TCA) to a final concentration of 20% for 15 min on ice. Upon centrifugation, precipitated proteins were washed with ethanol/ethyl acetate mixture (1∶1 v/v) three times. After the final washing, precipitates were incubated with pre-chilled 90% acetone for 30 min on ice. The acetone was then removed after centrifugation, the precipitates were allowed to air dry, and dissolved in rehydration buffer composed of 7 M urea, 2 M thiourea, 2% CHAPS, 0.5% ampholytes pH 3–10, and 0.002% (w/v) bromophenol blue. Between 30–35 µg of sample was applied to a 7 cm IPG strip. Strips were rehydrated overnight at room temperature. DTT (50 mM) was added to the rehydration buffer just before rehydrating the strips. The rehydrated IPG strips containing samples were then isoelectrically focused on an IPGphor (General Electric, Pittsburgh, PA). After isoelectric focusing, IPG strips were equilibrated with equilibration buffer I (2% SDS, 50 mM Tris-HCl pH 8.8, 6M urea, 30% (v/v) glycerol, 0.002% bromophenol blue, and 1% DTT) followed by equilibration buffer II (2% SDS, 50 mM Tris-HCl pH 8.8, 6M urea, 30%(v/v) glycerol, 0.002% bromophenol blue, and 2.5% iodoacetamide). Two-dimensional gel electrophoresis was performed on the strips to separate the proteins according to molecular mass. Gel staining and Western blot were performed using the procedure mentioned in total protein carbonylation. Densitometric intensity of the major carbonylated protein spots in the 2D-Western blots were divided by the corresponding protein spots in the coomassie blue staining gel to determine the carbonylation of corresponding proteins for each of the drug treatments.

### Protein identification

Five major proteins that showed significant differences in their contents and carbonylation in serum with drug treatment were selected for mass spectrometric analysis. Gel spots were excised from the gel and digested with sequencing grade modified trypsin (Promega Corporation, Madison, WI). Trypsin digested peptides were subjected to LC-MS/MS analysis using the Q-TOF LC/MS (Agilent Technologies, Santa Clara, CA). Peptide search and protein identification was performed using the Spectrum Mill MS Proteomic Workbench (Agilent Technologies, Santa Clara, CA).

### Statistics

Differences in cardiac lesion scores between groups were determined using the Kruskall-Wallis test (nonparametric analysis of variance). Differences in the tumor growth inhibition, γ-H2AX nuclear foci formation, hematology indices, active caspase-3, and serum levels of clinical chemistry analytes were compared using Student's *t*-test. The hematology indices and serum chemistry analytes were compared between groups using Bonferroni's multiple comparisons test. A value of *p*≤0.05 was considered statistically significant.

## Results

### Validation of the SHR/SST-2 animal model

**Figure 1:**
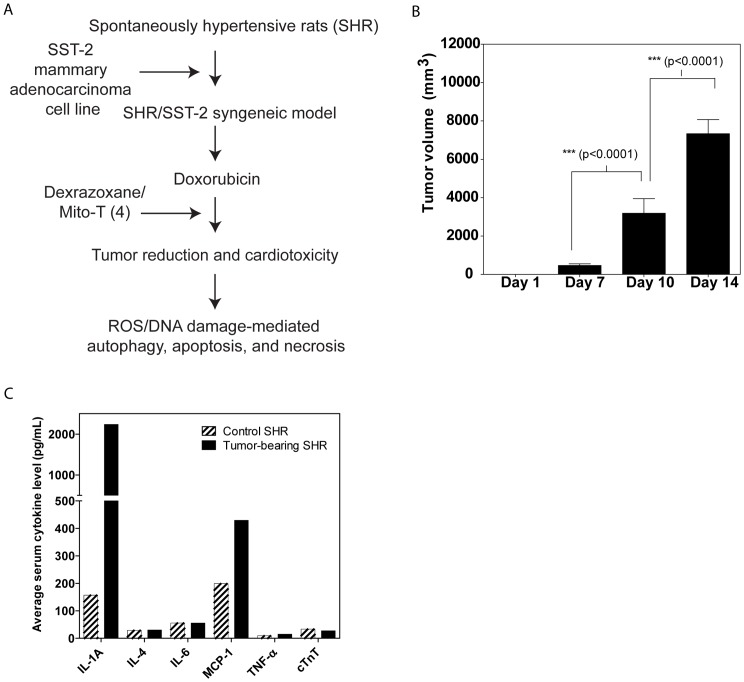
Optimization of the syngeneic SHR/SST-2 model. *A,* Study design for the optimization and application of the syngeneic SHR/SST-2 model. SHRs were subcutaneously implanted with exponentially-growing SST-2 breast cancer cells in their right mammary fat pads. Doxorubicin and dexrazoxane were used as control chemotherapeutic and chemoprotective agents, respectively. Mito-T (4) was tested as a chemoprotective agent. In addition to overall tumor reduction and cardiac lesions, protein oxidation, DNA oxidation, autophagy, apoptosis, and necrosis were measured as mechanistic endpoints. *B,* Tumors were measured at days 1, 7, 10, and 14. Results are expressed as tumor volume. *C,* Serum from control and tumor-bearing SHRs was compared for levels of inflammatory cytokines, IL-1A, IL-4, IL-6, MCP-1, and TNF-α. Cardiac troponin T (cTnT) was compared as an indicator of cardiac toxicity. Mean values from 30 animals per group are shown.

The right mammary fat pad of each SHR was injected with approximately 5×10^5^ SST-2 cells. Our experimental design is shown in Figure 1A. The resultant SST-2-bearing SHRs (60 females), referred to from here on as SHR/SST-2, showed rapid tumor uptake through day 14. Figure 1B shows the rate of tumor growth in the SHR/SST-2 animal model in the absence of any drug treatment. In addition to tumor growth, various hematological indices were examined to confirm an immune response to the tumor. Table S1 shows the hematological parameters that were assessed. An expected increase in monocytes was observed in the presence of tumor growth. Serum cytokine levels (Figure 1C) and other serum chemistry markers (Table S2B) were compared between control and tumor-bearing animals as indicators of general homeostasis following tumor implantation. Two cytokines, IL-1A and MCP-1, were increased in response to tumor implantation at day 14 (Figure 1C) which is in agreement with a tumor promoting role for IL-1A [39]. An increase in MCP-1 levels in response to tumor presence has also been previously reported [40]. The increase in IL-1A correlates with increased levels of monocytes that were observed in tumor-bearing rats. In addition to these hematological, immunological, and serum chemistry analyses, visual assessment of the tumor showed no obvious signs of necrosis at day 14 or tumor rejection, and several markers (serum chemistry, hematology indices, and weights) demonstrated that tumor uptake was normal. Table S1A shows the average initial and final body weights as well as heart weight at necropsy on day 14. The presence of the tumor alone appeared to have no significant impact on the heart weight of the animals. A slight increase in average heart size can be explained by the corresponding increase in the average animal weight over 2 weeks. Taken together these data indicate that the SHR model had adequate and successful uptake of the SST-2 tumor.

### Anti-tumor effects in the SHR/SST-2 animal model

**Figure 2:**
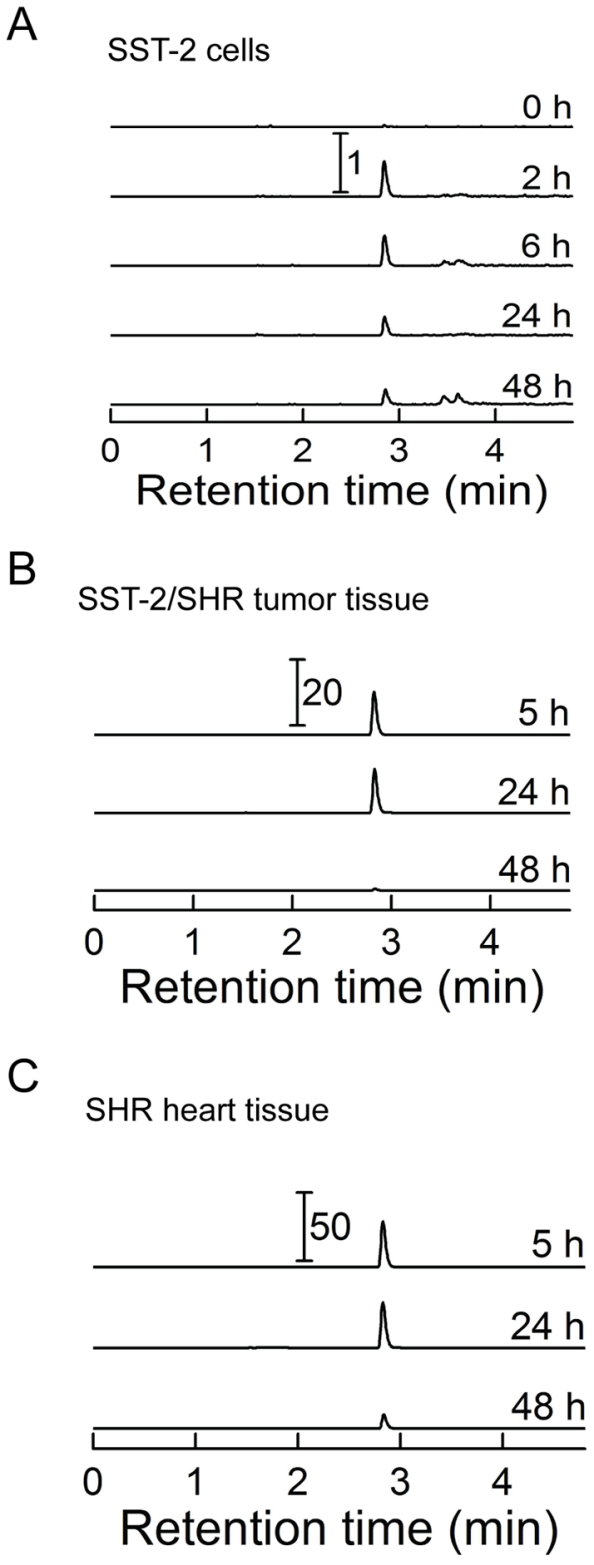
Accumulation of Mito-T (4) in mitochondria of heart and tumor cells and tissue. *A*, SST-2 cells were incubated with 10 μM Mito-T (4) for the indicated period of time and collected. Mito-T (4) was detected in isolated mitochondrial fractions by LC-MS/MS as described in the *Materials and Methods* section. *B*, SHR rats were implanted with SST-2 cells. Upon tumor growth of 1 cm^3^ over approximately 6 days, animals were treated with 25 mg/kg Mito-T (4). Necropsy was performed at 5, 24, and 48 h following administration of Mito-T (4) and tissue was harvested and frozen. Tumor tissue was homogenized and mitochondrial fraction assayed for Mito-T (4) content by LC-MS-MS. *C*, Same as panel B, but mitochondria from heart tissue were analyzed. All LC traces represent the MRM transition 489.0 → 474.2 and were scaled to the same protein level. The numbers with the scale bars indicate differences in signal intensities between the panels and relative to the signals from SST-2 cells (panel A).

Tumors were excised from 60 SHRs and were sub-cultured prior to further implantation in new SHRs for the drug studies. These new SHRs were then treated with the anthracycline chemotherapeutic, doxorubicin. Doxorubicin has classically been used to treat breast cancer, but its well-known cardiotoxicity is usually dose-limiting [7]. Other compounds utilized in our experiment include the iron chelator dexrazoxane and the recently developed redox-active nitroxide tempol conjugated to a triphenylphosphonium cation (TPP^+^), Mito-T (4). Mito-T (4) was tested in our experiment because evidence indicates that mitochondrial oxidative stress is a primary mechanism through which doxorubicin exerts its cardiotoxic effects [41]. To test if Mito-T (4) can accumulate in mitochondria, we analyzed mitochondrial fractions from SST-2 cancer cells in vitro, as well as from tumor and heart tissue in vivo, at different time points (up to 48 h) after administration of the compound (Figure 2). In all cases Mito-T (4) was detected in mitochondrial fractions and its content decreased significantly after 24 h in cultured cells and after 48 h in tissue samples in vivo. We observed a higher accumulation of Mito-T (4) in cardiac samples than in cancerous samples. The presented data confirm the ability of Mito-T (4) to accumulate in cardiac and cancerous mitochondria in vitro as well as in vivo.

Both dexrazoxane and Mito-T (4) were tested in the SHR/SST-2 system either alone or in combination with doxorubicin. We hypothesized that ROS and DNA damage induced by doxorubicin can result in cells activating survival pathways like autophagy and/or cell death pathways such as apoptosis and necrosis; hence we examined both cell survival and cell death pathways in both the tumor and heart.

Table S2 shows the results of heart and body weight analyses (Table S2A) as well as hematology (Table S2B) and serum chemistry (Table S2C) data from the animals exposed to the indicated drug with superscripted annotations noting significance as compared to a saline control. Doxorubicin in combination with Mito-T (4) induced a significant reduction in body weight in comparison to saline, doxorubicin, or Mito-T (4) alone. Weight loss has been previously observed in response to the parent compound of Mito-T (4), tempol, as well as with doxorubicin [12], [42]. No significant differences in the ratio of heart to body weight were observed with the drug treatments (Table S2A). In regards to hematology and serum chemistry, doxorubicin induced an anticipated decrease in albumin as well as increases in blood urea nitrogen and lipid levels (Table S2C). This was accompanied by increased immune response observed by increased levels of monocytes and granulocytes (Table S2B). These markers have been demonstrated previously to be broad indicators of renal and cardiac stress [43]. Low dose Mito-T (4) appears to have been able to reduce some of the acute toxicity indices of doxorubicin, specifically the lowered glucose in the serum, and altered lymphocyte and granulocyte levels in the blood. Dexrazoxane exhibited rescue of serum albumin, alanine l-transferase, total bilirubin, glucose, and lipid levels. The lower dose of Mito-T (4) was better able to normalize the hematological indices than the higher dose. Overall, dexrazoxane appeared to mitigate the hematology and serum chemistry markers of overt toxicity to a greater extent than Mito-T (4).

**Figure 3:**
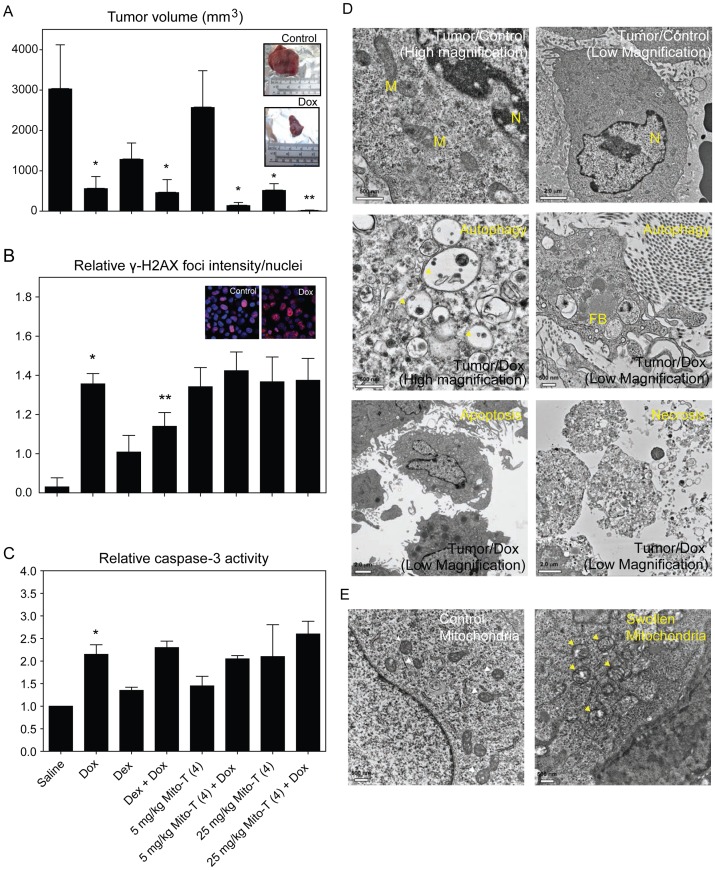
Tumor growth and response to chemotherapeutic and chemoprotective agents in SHR/SST-2 animals. *A,* SHRs were implanted with SST-2 cells and 24 h later were administered either doxorubicin (10 mg/kg), dexrazoxane (50 mg/kg), Mito-T (4) (5 or 25 mg/kg), a combination of doxorubicin and dexrazoxane, or a combination of doxorubicin and Mito-T (4). Each treatment group consisted of 10 animals. The mean tumor volumes (mm^3^) measured 14 days after drug treatment are shown for each treatment group. The two *inset* images show representative excised tumors from saline and doxorubicin-treated SHR/SST-2 animals. *B*, DNA damage by doxorubicin and chemoprotective agents was measured in tumor cells by confocal microscopic detection of γ-H2AX foci. Average foci intensity was measured in at least 100 cells per drug treatment observed from at least 10 representative fields. The data are represented as fold increase over no treatment tumor cells (Control). *C*, Active caspase-3 levels were assessed as a marker of apoptosis induction. Average fold increase is shown over saline control tumor tissue samples. * =  statistically significant compared to saline and ** = statistically significant compared to doxorubicin (A–C). *D,* Transmission electron microscopy analysis of tumor samples from rats exposed to doxorubicin showing the formation of autophagic vacuoles (Autophagy panel), or nuclear condensation and membrane blebbing (Apoptosis panel), and membrane breakdown (Necrosis panel). Saline treated (Tumor/Control panels) are shown as controls. Representative low- and high-magnification images for the control and autophagic samples are shown for clarity. Mitochondria (labeled M), nucleus (N), and fat bodies (FB) are labeled within the images. *E,* Swollen mitochondria following doxorubicin treatment (right) are indicated with yellow arrows in comparison to mitochondria from saline-treated animals (left) indicated with white arrows. Scale bars and magnification are indicated on each panel. Quantification of the mitochondrial cross-section areas is provided in Figure S2.

Figure 3A shows the antitumor activity of dexrazoxane and Mito-T (4), either alone or in combination with doxorubicin. As expected, the front-line agent against breast cancer, doxorubicin, reduced tumor size significantly over the 2-week experimental period. Dexrazoxane alone reduced the average tumor size, though not significantly, and it did not interfere with doxorubicin-induced tumor reduction when tested in combination. Mito-T (4) alone caused significant tumor reduction and appeared to increase the anti-tumor effect of doxorubicin at both dose levels (5 mg/kg and 25 mg/kg). The inset images of Figure 3A show representative excised tumors from animals treated with saline or doxorubicin.

The induction of DNA damage in tumor tissue was assessed using the γ-H2AX assay to measure the average number of DNA double-strand breaks (DSBs), expressed as the average γ-H2AX foci intensity per nucleus in Figure 3B [44]. Doxorubicin induced significant DNA damage in the tumor tissue by day 14. Dexrazoxane alone also increased the levels of γ-H2AX in tumor cells, while Mito-T (4) alone appeared to induce levels of DNA DSBs in the tumor comparable to that of doxorubicin. The inset images in Figure 3B show representative γ-H2AX staining in saline versus doxorubicin treated tumor tissues. The combination of doxorubicin with dexrazoxane had lower levels of γ-H2AX than the combination with Mito-T (4), although both agents were able to sustain tumor size reduction as seen in Figure 3A. The presence of increased DNA DSBs as shown by γ-H2AX staining has been shown to be linked to increased oxidative stress [45]. The presence of single-strand DNA breaks (SSBs) has also been linked to increased oxidative stress in cells. Data from an alkaline comet assay, which assesses both DSBs and SSBs, also were in agreement with the γ-H2AX findings (comet data not shown).

Figure 3C shows the fold increase in active caspase-3 that was seen in the tumors of animals treated with the various drugs. Doxorubicin-treated tumor lysates displayed an increased caspase-3 activity compared to saline-treated tumor samples. Mito-T (4) alone appeared to induce similar levels of caspase-3 as dexrazoxane, both at levels slightly above saline. The combination of either dexrazoxane or Mito-T (4) with doxorubicin did not diminish the apoptotic activity of doxorubicin. Thus, Mito-T (4) and dexrazoxane were able to sustain the DNA damage, apoptosis, and tumor reduction of doxorubicin in tumor cells.

In order to provide a qualitative assessment of cellular response mechanisms as well as mitochondrial health in response to doxorubicin in our new animal model, transmission electron microscopy (TEM) analysis was performed on tumor tissues. Figure 3D shows tumor tissue in control versus doxorubicin treated animals at both high and low magnification. The top row contains high (left) and low (right) magnification images of a control tumor cell (left) with an apparently normal nucleus (N) and several mitochondria (M). The second row features high (left) and low (right) magnification images of a tumor cell from a doxorubicin treated animal in which the cell is evidently autophagic. Vacuoles shown in the images illustrate that the cell is undergoing typical autophagosome-mediated recycling. The bottom row of Figure 3D shows low magnification TEM demonstrating apoptosis (left) and necrosis (right) in doxorubicin-treated tissue. Figure 3E also shows mitochondrial swelling in doxorubicin-treated tumor tissue compared to saline control. Arrows indicate mitochondria that are clearly swollen in the drug-treated cells, confirming mitochondrial distress. A quantitative comparison of the distribution of mitochondrial cross-sectional area also indicated mitochondrial swelling to be statistically significant in treated tumor cells (Figure S2). Thus, we are able to confirm DNA damage, mitochondrial damage, apoptosis, autophagy, and necrosis in tumor tissue exposed to doxorubicin. We also demonstrate that both dexrazoxane and Mito-T (4) enhance the antitumor effects of doxorubicin.

### Cardiotoxicity and autophagy in the animal model

**Figure 4:**
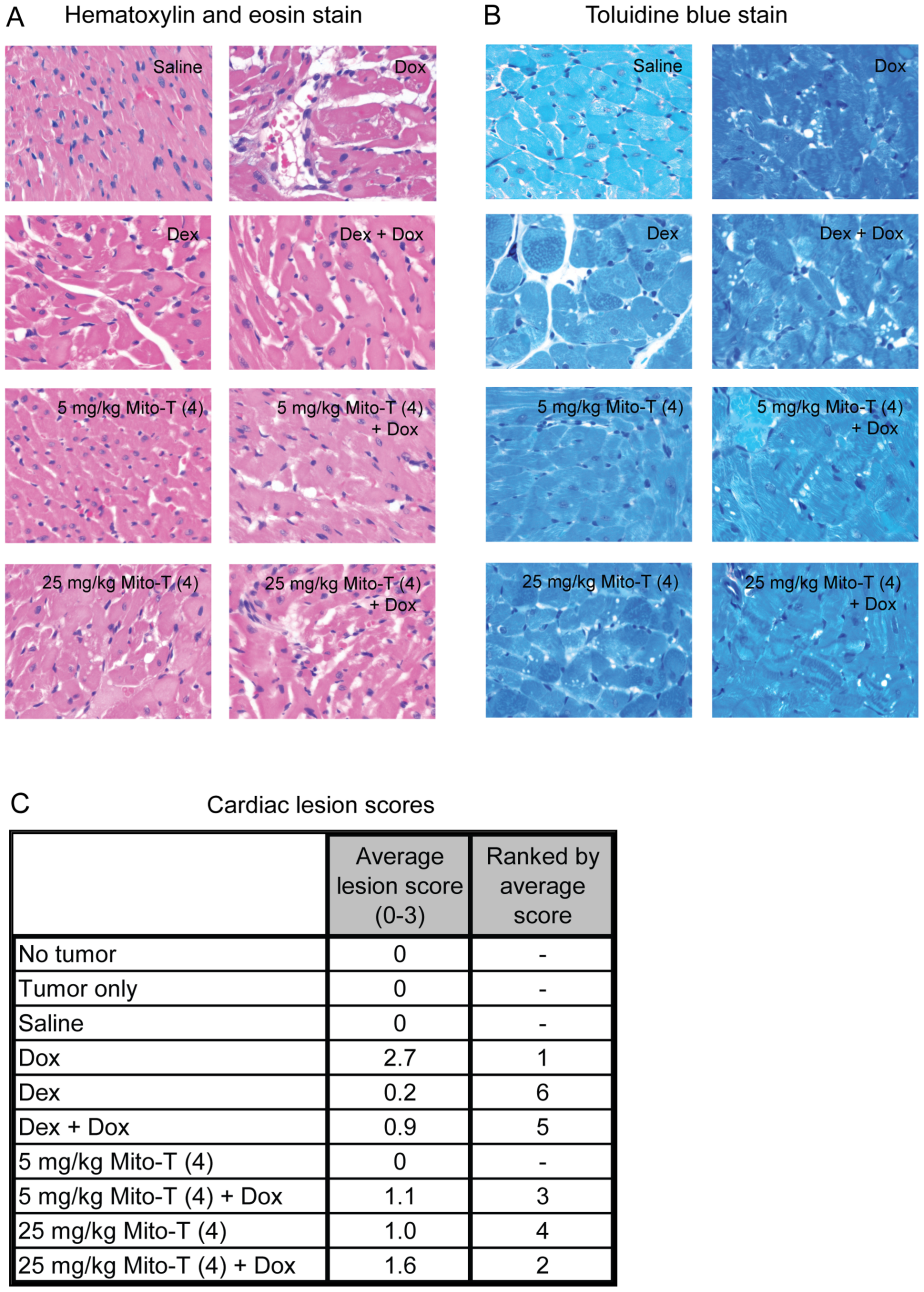
Cardiotoxicity and cardioprotection in SHR/SST-2 animals. *A–B*, Representative photomicrographs of cardiac histological sections are shown following either hematoxylin and eosin staining (panel A) or toluidine blue staining (panel B). *C*, Numerical cardiomyopathy scores (maximum severity score  = 3) are shown following analysis of each treatment group (n = 5). Please refer to the methods and results sections for descriptions of the scoring method and treatment-induced cardiomyopathy, respectively.

Cardiotoxicity in our animal model was assessed by histopathological evaluation of cardiac sections with both hematoxylin and eosin (Figure 4A) and toluidine blue staining (Figure 4B). Cardiac sections were scored for the presence of lesions. The average score and rank by average score are shown in Figure 4C. As expected, doxorubicin induced the highest number of lesions, which were mitigated by the addition of the known cardioprotectant dexrazoxane. Notably, Mito-T (4) also appeared capable of mitigating the cardiotoxicity of doxorubicin. Thus, in addition to enhancing doxorubicin-mediated antitumor activity, Mito-T (4) appeared to exhibit a cardioprotective effect in response to the cardiotoxicity of doxorubicin.

**Figure 5:**
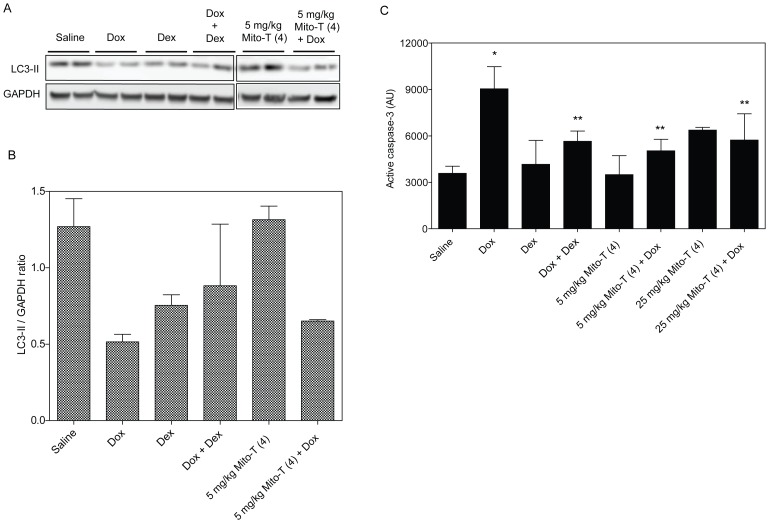
Autophagy and apoptosis in cardiac tissue after cardiotoxic and cardioprotective treatment. *A,* Western blotting of autophagy-indicative lipidated LC-3 II protein in cardiac tissue samples. Protein extracts analyzed for LC3-II from two representative SHR/SST-2 animals per treatment group are shown. *B,* The mean LC3-II/GAPDH ratio in each treatment group by Western blotting analysis is shown. Quantitation was performed by densitometric analysis of the LC3-II bands from panel A. GAPDH protein levels were used as a loading control. *C*, Paraffin-embedded cardiac tissue were stained with anti-active caspase-3 antibody. The intensity of HRP-tagged secondary antibody was quantified as an indication of active caspase-3 using the ScanScope software. Mean intensities are shown in the graph and derived from at least 10 images per animal (5 animals per group). * =  statistically significant compared to saline and ** = statistically significant compared to doxorubicin.

One hypothesis for cellular protection during oxidative stress is that increased autophagy might serve to protect tissues from other forms of cell death such as apoptosis and necrosis and may help to preserve tissue integrity. We therefore looked at the overall levels of autophagy in the cardiac tissues of drug-treated animals. Figure 5A shows representative Western blotting of the common autophagy marker LC3-II from two representative heart tissue lysates per treatment condition. The average LC3-II protein level relative to a GAPDH loading control in cardiac tissues of test animals is shown in Figure 5B. These data demonstrate that doxorubicin causes a decrease in pro-survival autophagy in the heart compared to the saline control. However, the combinations of doxorubicin with dexrazoxane, or to a lesser extent Mito-T (4), show higher levels of autophagy when compared to doxorubicin alone.

While Western blotting showed that doxorubicin appeared to reduce the level of autophagy in the heart, it appeared to increase the level of apoptosis. Figure 5C shows active caspase-3 levels as indicated by the intensity of HRP-tagged secondary antibody. The data show an increase in apoptosis in response to doxorubicin treatment that is not mirrored in the other drug-treated tissues. We note that Mito-T (4) alone showed increased caspase-3 at the 25 mg/kg dose as a single agent, while the combination with doxorubicin was lower than doxorubicin alone. These results may indicate a shift in the balance of autophagy and apoptosis that would result in cardiotoxicity in doxorubicin-treated animals. However, the induction of apoptosis together with a reduction in autophagy may be mitigated by the presence of the antioxidants dexrazoxane and Mito-T (4).

### DNA and protein oxidation in SHR/SST-2 serum

**Figure 6:**
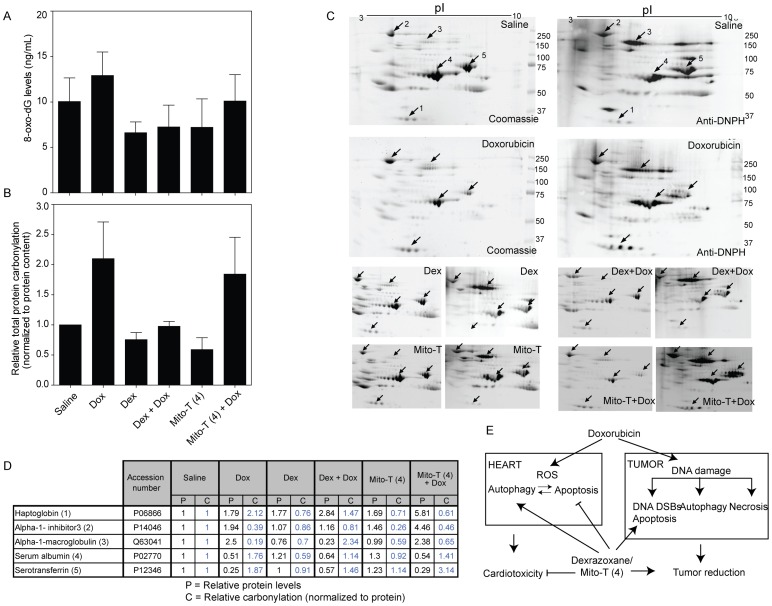
Oxidative damage to DNA and proteins in SHR serum. *A*, DNA oxidative modification as measured by 8-hydroxy-2′-deoxyguanosine levels in SHR serum using an ELISA assay. Average from at least 4 animals per group is shown *B*, Protein carbonylation of serum was measured as an indication of protein oxidation. Serum samples (5 µg) from drug treated groups of animals were derivatized with DNP and electrophoresed on two parallel gels for each experiment. One gel was stained with coomassie blue to determine total protein and the other gel was used for Western blotting. Carbonylated proteins were detected using anti-DNPH antibody. Total serum protein carbonylation levels shown are relative values compared to total protein carbonylation of the saline treated samples normalized to 1. Quantitation was performed by densitometric analysis of the whole lanes for gel staining and Western blot. *C*, Representative 2D-gels from serum samples from drug treated, tumor bearing SST-2 rats are shown. Left panels for each sample represent the coomassie gel staining and right panels represent the Western blot analysis using anti-DNP antibody. Spots labeled 1–5 were the major serum proteins that exhibited significant changes in carbonylation and concentration comparing saline and doxorubicin. The top four panels show the complete gel data for saline and doxorubicin samples, while the bottom eight panels show the significant spots from other drug treated samples. The carbonylated proteins with the most appreciable changes were identified by LC-MS/MS analysis as haptoglobin (1), alpha-1-inhibitor 3 (2), alpha-1-macroglobulin (3), serum albumin (4), and serotransferrin (5). *D*, Table shows relative protein content and specific protein carbonylation in drug treated SHRs compared to saline treated rats determined by 2D-gel electrophoresis from panel C. Data are representative of three animals analyzed per treatment group. *E,* Proposed model for pro-autophagic and anti-apoptotic mechanism of action for dexrazoxane and Mito-T (4) in combination with doxorubicin.

We next examined protein and DNA oxidation as potential mechanisms though which cardioprotection or chemotherapeutic outcome may be affected. Serum was chosen for its relative ease of extraction in case clinically useful biomarkers were identified in this study. In DNA, guanine is the nucleotide most prone to oxidative damage. However, during DNA repair, oxidatively modified 8-hydroxy-2′-deoxyguanosine (8-oxo-dG) is removed. Therefore, the amount of free 8-oxo-dG in the serum can be used as an indicator of oxidative stress. Figure 6A shows a slight increase in 8-oxo-dG levels in serum after doxorubicin treatment. This effect appears to be mitigated with the addition of either dexrazoxane or Mito-T (4), although the protective effect appeared to be greater by dexrazoxane (not statistically significant). We note that the basal levels of 8-oxo-dG in the saline-treated animals were high and attribute this to the presence of the tumor.

ROS-induced carbonylated proteins were DNP-derivatized and analyzed by Western blotting using anti-DNP antibody (Figure 6B). Total protein carbonylation in serum of doxorubicin-treated SHRs was approximately two-fold higher than saline-treated SHRs. Dexrazoxane and Mito-T (4) alone did not significantly alter the total protein carbonylation. Consistent with an antioxidant cardioprotective mechanism for dexrazoxane, serum from doxorubicin and dexrazoxane-treated animals showed a significant decrease in total carbonylation when compared to doxorubicin alone. Mito-T (4) in combination with doxorubicin showed a slight but not-statistically significant decrease in carbonylation. To further dissect the specific proteins that are oxidized and the potential mechanistic differences between dexrazoxane and Mito-T (4), the carbonylation of individual proteins were assessed using 2D-gel electrophoresis. Using the 2D-gel profiles, five major proteins (haptoglobin, alpha-1-inhibitor 3, alpha-1 macroglobulin, serum albumin, and serotransferrin) were identified as having significantly changed protein levels and extent of carbonylation (Figure 6C, 6D). Following doxorubicin treatment, albumin and serotransferrin protein levels decreased in the serum and displayed significant increases in their carbonylation (relative to protein levels) compared to saline treated animals. Alpha-1-macroglobulin and alpha-1-inhibitor 3 protein levels increased while exhibiting a decrease in carbonylation levels. Doxorubicin in combination with Mito-T (4) showed similar protein levels for albumin, serotransferrin, and alpha-1-macroglobulin but haptoglobin and alpha-1-inhibitor 3 levels were elevated. Doxorubicin-induced carbonylation was decreased on haptoglobin and albumin in the serum samples. Dexrazoxane in combination with doxorubicin displayed reduced protein carbonylation for each of the proteins except alpha-1-macroglobulin compared to doxorubicin-treated animals. Mito-T (4) did not comparably reduce the oxidative damage caused by doxorubicin in these plasma proteins. The slightly different anti-oxidant properties of mitochondrially targeted Mito-T (4) in heart tissues (Figure 5) compared to serum (Figure 6) indicates that SOD mimetic Mito-T (4) may act differently at cellular and extracellular levels than the iron chelating dexrazoxane.

## Discussion

In this study, we endeavored to develop an animal model to simultaneously investigate the antitumor and cardiotoxic activities of drugs. The need for a model of this sort with a fully functional immune system has been demonstrated by the trend of cancer chemotherapies to induce cardiotoxicity in patients, regardless of drug class. An animal model could be used to test both anticancer efficacy and cardiotoxicity, as well as to investigate the cellular mechanisms behind these effects. In order to investigate cardioprotective and antitumor effects in the same animal model, we examined the acute toxicity from a cardiotoxic dose of doxorubicin following implantation of SHR with a syngeneic breast cancer cell line SST-2 [21], [46]. The known cardiotoxic chemotherapeutic doxorubicin reduced tumor size and induced significant cardiac toxicity in our animals. Likewise, the iron chelator dexrazoxane, previously shown to mitigate the cardiotoxicity of doxorubicin, performed as expected. In addition to control compounds, we also tested the novel antioxidant Mito-T (4) and found that it has both anticancer and cardioprotective properties, distinct from dexrazoxane. These results indicate that further work on the mechanisms and utility of Mito-T (4) in the breast cancer model is warranted. Our novel findings of autophagy, protein oxidation, and identification of specific serum proteins that are oxidized under cardiotoxic conditions provide potential leads for subsequent work on the roles of these proteins in cardiotoxicity and evaluation of their potential as clinical biomarkers.

In addition to helping to identify promising compounds for future study, tissue analysis of our animal model has lead to a model for the cellular interplay between antitumor activity and cardiotoxicity in response to doxorubicin. Doxorubicin is known to be both redox active and a potent inducer of DNA damage. In separate studies, the oxidative stress and DNA damaging activities of doxorubicin have been shown to result in the induction of apoptosis, autophagy, and necrosis [47], [48]. Our studies provide concurrent and quantitative analysis of autophagy and apoptosis in both cardiac and tumor tissue, while providing qualitative evidence of necrosis in the tumor tissue. To our knowledge, this is the first reported simultaneous measurement of autophagy and apoptosis in heart and tumor tissues exposed to doxorubicin in a physiologically-relevant animal model. In the heart, doxorubicin appeared to cause a shift from autophagy to apoptosis, resulting in cardiotoxicity (Figure 6E). Similar to the histopathology analysis, TEM imaging revealed an increased number of lesions and less regular striation patterns in doxorubicin-treated heart tissue (Figure S3). Dexrazoxane and Mito-T (4) inhibit apoptosis and induce the cell-protective autophagic pathway, thus mitigating cardiac damage. In the tumor on the other hand, dexrazoxane and Mito-T (4) induce apoptosis leading to increased tumor reduction in combination with doxorubicin. Future work is required to determine why different effects are found in different tissues, but it is tempting to speculate that the higher endogenous baseline level of ROS in the tumor might shift the balance toward cell death in response to oxidative stress induced by doxorubicin. We confirmed the anticancer potential of Mito-T (4) as a single agent in the NCI-60 panel (Figure S4) and found that leukemia and breast cancer cell line groups were more sensitive in the SRB assay than other cell lines. Mito-T (4) resulted in a weight loss at the high dose of 25 mg/kg and when combined with doxorubicin at either dose tested (Table S2A). This loss in weight is in agreement with previous findings with tempol in C3H and CBA mice that showed chronic supplementation of tempol reduced body weight without toxicity while decreasing cancer and extending survival of mice exposed to nonlethal total body radiation [49].

We also observed that dexrazoxane and Mito-T (4) exhibited different mechanisms of cytotoxicity. Specifically, Mito-T (4) alone showed more γ-H2AX-marked DNA double-stranded breaks than dexrazoxane (Figure 3B) in the presence of comparable apoptosis in the tumor (Figure 3C). Mito-T (4) alone had less of an effect on autophagy in the cardiac tissue than dexrazoxane (Figure 5B) while providing comparable protection or greater combined autophagy with doxorubicin (Figures 4 and 5B). The enhanced autophagy as a mechanism for cardioprotection is in agreement with other studies using doxorubicin or other models of cardiac dysfunction [50], [51]. Further studies are needed to determine the exact molecular differences in the autophagic and apoptotic mechanisms of dexrazoxane and Mito-T (4). Tempol conjugated with TPP^+^ could be an effective SOD mimetic based on previous reports on tempol and other nitroxides [52], [53]. Due to the increased negative membrane potential in tumor cells, the cationic nitroxide Mito-T (4) accumulates in tumor mitochondria as confirmed by our studies (Figure 2). Previous publications with the mitochondrially-targeted nitroxide (Mito-CP) and quinone (Mito-Q) also reported significant and selective toxicity in colon, breast, and lung cancer cell lines as well as animal models [24], [54].

Levels of 8-oxo-dG in serum increased after doxorubicin treatment, which suggests an increase in oxidative damage. As expected, treatment with dexrazoxane or Mito-T (4) lessened the oxidative DNA damage caused by doxorubicin. Although these data show detectable levels of 8-oxo-dG in serum, we can speculate that similar relative patterns of oxidative DNA damage are induced in heart and tumor tissues after doxorubicin treatment. Therefore, dexrazoxane and Mito-T (4) decreased oxidative DNA damage caused by doxorubicin in heart tissue and this might reduce apoptosis of cardiomyocytes (proposed mechanism in Figure 6E). In contrast, tumor tissue should have higher endogenous levels of DNA damage; hence additional DNA damage after treatment may induce apoptosis. The exact mechanisms by which dexrazoxane and Mito-T (4) trigger DNA damage need to be examined further. For instance, whether Mito-T (4) directly targets the DNA synthesis or repair machinery, or whether the γ-H2AX and 8-oxo-dG signals are indicative of secondary DNA lesions from free radicals or metabolites.

Plasma proteins such as albumin, transferrin, and ceruloplasmin act as anti-oxidants in plasma preventing iron and copper mediated generation of hydroxyl radicals [55]. In addition, albumin effectively scavenges free radicals. A decrease in albumin is related to coronary heart disease and heart failure [56], and is in agreement with our observation of decreased serum albumin under cardiotoxic conditions (Table S2C and Figure 6D). While serotransferrin prevents the iron-mediated formation of free radicals, direct correlation of serotransferrin concentration and risk of cardiovascular diseases has not yet been reported. Treatment of SHR with doxorubicin decreased the albumin and transferrin levels in serum and increased the extent of carbonylation of both proteins compared to saline treated SHR (Figure 6C and 6D). When serum albumin levels decrease, concentrations of high molecular weight proteins such as macroglobulin and α1-inhibitor 3 increase to maintain total plasma protein concentration [57]. The increase in carbonylation and decrease in concentration of anti-oxidant plasma proteins in response to doxorubicin treatment indicate that the carbonylation of these proteins might be indicative of oxidative stress and cardiotoxicity, and thereby used as biomarkers of such damage. These plasma proteins show different responses to doxorubicin treatment in combination with Mito-T (4) or dexrazoxane, and thus follow-up studies are required to understand the distinct antioxidant properties of these drugs in the context of the oxidation of individual serum proteins. Although we and others have demonstrated the mitochondrial localization of TPP-conjugated compounds in cells, the relative intracellular accumulation of Mito-T (4) compared to other TPP-conjugated compounds and the direct impact of sub-cellular Mito-T (4) concentrations on the cardioprotective and anticancer outcomes in whole animal systems are not clear [31]. We also note that while the SHR/SST-2 animal model provides advantages of a stable phenotype and well-established cardiotoxicity profile for mechanistic studies, the effects of redox-active agents under normotensive conditions in animals or in humans require further comparative studies. In summary, the SHR/SST-2 model not only offers advantages for testing novel anticancer and cardioprotective therapeutics and biomarkers in a single, established model, but offers the potential to answer a number of unresolved questions in the field of cardiotoxicity by oncology agents.

## Supporting Information

Figure S1 **Scheme for synthesis of Mito-T (4).** *A*, Mito-T (4) synthesis involved first the synthesis of Tempol-bromobutylether and then reacting this with triphenylphosphine to obtain Mito-T (4). *B*, Purity of the product was ascertained by performing HPLC and LC/MS (mass  = 489).
(TIF)

Figure S2 **Comparison of mitochondrial cross-sections.** The cross-sections showing mitochondrial swelling in Figure 3E were analyzed for quantitative differences in mitochondrial cross-section area. The area of 350 tumor cell mitochondria cross-sections from a representative animal tissue sample was determined from several random images of each sample (control and doxorubicin treated). The histograms shown indicate that most mitochondria cross-sections fall into the same range in both samples (from 0.1 to 0.2 μm^2^) but in doxorubicin treated tumor cells a significant amount of cross-sections also fall in a larger size range (from 0.2 to 0.4 μm^2^). The change in size distribution to larger size range of mitochondrial cross-sections is indicative of mitochondrial swelling. The median, mean, and standard deviation values were 0.14, 0.16+0.09 μm^2^ and 0.21, 0.23+0.11 μm^2^ for control and doxorubicin treated, respectively. The difference is statistically significant with a p<0.0001 using a one-tail, non-parametric Mann-Whitney test. The dotted lines on the graphs indicate the median value of the data set.
(TIF)

Figure S3 **Ultrastructural analysis of autophagy in the cardiac tissue.** Transmission electron microscopy was used to confirm autophagy in the heart tissue from SHR/SST-2 cells exposed to a cardiotoxic dose of doxorubicin. Low and high-magnification images are shown for clarity. Representative images of normal cells from saline treated tissue and damaged/autophagic cells from doxorubicin-treated tissue are shown. While autophagy was observed by TEM analysis in both saline and doxorubicin-treated heart tissue, a relative quantitative assessment could not be accurately made by TEM analysis due to the low occurrence of autophagic fraction of cells in the sarcomere matrix made up of myocytes and myofibrils.
(TIF)

Figure S4 **Anticancer activity of Mito-T (4)** *A,* Graph illustrating the NCI-60 anticancer activity profile for Mito-T (4) as tested by the SRB assay in the NCI-60 anticancer drug screen cell line panel. Z-Score values for each cell line are plotted relative to the mean across all cell lines. Bars towards the right of the mean, such as the breast cancer and leukemia groups, are indicative of sensitivity relative to the mean across all cell lines. *B*, Dose response curves for Mito-T (4) in the breast cancer and leukemia groups of cell lines. Log base 10 of the molar concentrations used in the SRB assay are plotted against the percentage growth in cells over 72 h.
(TIF)

Table S1 **Heart/body weights, hematology indices, and serum chemistry in SHR with or without SST-2 cells.** *A,* Control and tumor-bearing SHR were weighed before and 14 days after SST-2 implantation. The hearts were weighed post-necropsy on day 14. *B,* Whole blood from non-tumor bearing and tumor-bearing SHR was analyzed for white blood cell (WBC), lymphocyte, monocyte, granulocyte, hematocrit, hemoglobin, and platelet numbers. These data were compared to the expected values in SHR animals shown in the far right column. *C,* Serum from non-tumor bearing (Control SHR) and tumor-bearing SHRs was analyzed post-necropsy to assess the impact of tumor growth on animal health. The table shows the mean serum concentrations of albumin (ALB), alanine aminotransferase (ALT), total bilirubin (TBIL), blood urea nitrogen (BUN), creatinine (CRE), and glucose (GLU).
(TIF)

Table S2 **Heart/body weights, hematology indices, and serum chemistry in SHR with SST-2 cells after drug treatment.** The analysis of weights, hematology and serum chemistry were performed similar to Table S1. The animals were treated as indicated in the first column of each set of data. *A*, Ratios of final to initial body weight and heart to body weight. *B*, Whole blood analyses. *C*, Serum chemistry.
(TIF)

## References

[1] Albini A, Pennesi G, Donatelli F, Cammarota R, De Flora S, et al (2010) Cardiotoxicity of anticancer drugs: the need for cardio-oncology and cardio-oncological prevention. J Natl Cancer Inst 102: 14–25. 20007921 10.1093/jnci/djp440 PMC2802286

[2] Cardinale D, Colombo A, Torrisi R, Sandri MT, Civelli M, et al (2010) Trastuzumab-induced cardiotoxicity: clinical and prognostic implications of troponin I evaluation. J Clin Oncol 28: 3910–3916. 20679614 10.1200/JCO.2009.27.3615

[3] Mellor HR, Bell AR, Valentin JP, Roberts RR (2011) Cardiotoxicity associated with targeting kinase pathways in cancer. Toxicol Sci: 14–32. 10.1093/toxsci/kfq378 21177772

[4] Ferrans VJ, Clark JR, Zhang J, Yu ZX, Herman EH (1997) Pathogenesis and prevention of doxorubicin cardiomyopathy. Tsitologiia 39: 928–937. 9505340

[5] Levine RL, Stadtman ER (2001) Oxidative modification of proteins during aging. Exp Gerontol 36: 1495–1502. 11525872 10.1016/s0531-5565(01)00135-8

[6] Shacter E, Williams JA, Lim M, Levine RL (1994) Differential susceptibility of plasma proteins to oxidative modification: examination by western blot immunoassay. Free Radic Biol Med 17: 429–437. 7835749 10.1016/0891-5849(94)90169-4

[7] Olson RD, Boerth RC, Gerber JG, Nies AS (1981) Mechanism of adriamycin cardiotoxicity: evidence for oxidative stress. Life Sci 29: 1393–1401. 7029182 10.1016/0024-3205(81)90001-1

[8] Herman EH, Zhang J, Chadwick DP, Ferrans VJ (2000) Comparison of the protective effects of amifostine and dexrazoxane against the toxicity of doxorubicin in spontaneously hypertensive rats. Cancer Chemother Pharmacol 45: 329–334. 10755322 10.1007/s002800050048

[9] Moghrabi A, Levy DE, Asselin B, Barr R, Clavell L, et al (2007) Results of the Dana-Farber Cancer Institute ALL Consortium Protocol 95–01 for children with acute lymphoblastic leukemia. Blood 109: 896–904. 17003366 10.1182/blood-2006-06-027714 PMC1785142

[10] Bryant HE, Schultz N, Thomas HD, Parker KM, Flower D, et al (2005) Specific killing of BRCA2-deficient tumours with inhibitors of poly(ADP-ribose) polymerase. Nature 434: 913–917. 15829966 10.1038/nature03443

[11] Doggrell SA, Brown L (1998) Rat models of hypertension, cardiac hypertrophy and failure. Cardiovasc Res 39: 89–105. 9764192 10.1016/s0008-6363(98)00076-5

[12] Rao VA, Zhang J, Klein SR, Espandiari P, Knapton A, et al (2011) The iron chelator Dp44mT inhibits the proliferation of cancer cells but fails to protect from doxorubicin-induced cardiotoxicity in spontaneously hypertensive rats. Cancer Chemother Pharmacol 68: 1125–1134. 21373894 10.1007/s00280-011-1587-y

[13] Lindpaintner K, Kreutz R, Ganten D (1992) Genetic variation in hypertensive and ‘control’ strains. What are we controlling for anyway? Hypertension 19: 428–430. 1568759 10.1161/01.hyp.19.5.428

[14] Folkow B (1993) Early structural changes in hypertension: pathophysiology and clinical consequences. J Cardiovasc Pharmacol 22 Suppl 1S1–6. 7507535

[15] Herman EH, Ferrans VJ (1998) Preclinical animal models of cardiac protection from anthracycline-induced cardiotoxicity. Semin Oncol 25: 15–21. 9768819

[16] Herman EH, Zhang J, Lipshultz SE, Rifai N, Chadwick D, et al (1999) Correlation between serum levels of cardiac troponin-T and the severity of the chronic cardiomyopathy induced by doxorubicin. J Clin Oncol 17: 2237–2243. 10561281 10.1200/JCO.1999.17.7.2237

[17] Herman EH, el-Hage A, Ferrans VJ (1988) Protective effect of ICRF-187 on doxorubicin-induced cardiac and renal toxicity in spontaneously hypertensive (SHR) and normotensive (WKY) rats. Toxicol Appl Pharmacol 92: 42–53. 3124293 10.1016/0041-008x(88)90226-8

[18] McDonnell AM, Nowak AK, Lake RA (2011) Contribution of the immune system to the chemotherapeutic response. Semin Immunopathol 33: 353–367. 21274535 10.1007/s00281-011-0246-z

[19] Nagayasu H, Hamada J, Kawano T, Konaka S, Nakata D, et al (1998) Inhibitory effects of malotilate on invasion and metastasis of rat mammary carcinoma cells by modifying the functions of vascular endothelial cells. Br J Cancer 77: 1371–1377. 9652751 10.1038/bjc.1998.229 PMC2150200

[20] Hamada J, Nagayasu H, Takayama M, Kawano T, Hosokawa M, et al (1995) Enhanced effect of epidermal growth factor on pulmonary metastasis and in vitro invasion of rat mammary carcinoma cells. Cancer Lett 89: 161–167. 7889524 10.1016/0304-3835(95)03686-q

[21] Hamada J, Takeichi N, Kobayashi H (1987) Inverse correlation between the metastatic capacity of cell clones derived from a rat mammary carcinoma and their intercellular communication with normal fibroblasts. Jpn J Cancer Res 78: 1175–1178. 3121552

[22] Yuhki N, Hamada J, Kuzumaki N, Takeichi N, Kobayashi H (1986) Metastatic ability and expression of c-fos oncogene in cell clones of a spontaneous rat mammary tumor. Jpn J Cancer Res 77: 9–12. 3082820

[23] Kimmelman AC (2011) The dynamic nature of autophagy in cancer. Genes Dev 25: 1999–2010. 21979913 10.1101/gad.17558811 PMC3197199

[24] Rao VA, Klein SR, Bonar SJ, Zielonka J, Mizuno N, et al (2010) The antioxidant transcription factor Nrf2 negatively regulates autophagy and growth arrest induced by the anticancer redox agent mitoquinone. J Biol Chem 285: 34447–34459. 20805228 10.1074/jbc.M110.133579 PMC2966059

[25] Lipshultz SE, Scully RE, Lipsitz SR, Sallan SE, Silverman LB, et al (2010) Assessment of dexrazoxane as a cardioprotectant in doxorubicin-treated children with high-risk acute lymphoblastic leukaemia: long-term follow-up of a prospective, randomised, multicentre trial. Lancet Oncol 11: 950–961. 20850381 10.1016/S1470-2045(10)70204-7 PMC3756093

[26] Kruit WH, Punt KJ, Goey SH, de Mulder PH, van Hoogenhuyze DC, et al (1994) Cardiotoxicity as a dose-limiting factor in a schedule of high dose bolus therapy with interleukin-2 and alpha-interferon. An unexpectedly frequent complication. Cancer 74: 2850–2856. 7954247 10.1002/1097-0142(19941115)74:10<2850::aid-cncr2820741018>3.0.co;2-t

[27] Hasinoff BB, Chee GL, Thampatty P, Allan WP, Yalowich JC (1999) The cardioprotective and DNA topoisomerase II inhibitory agent dexrazoxane (ICRF-187) antagonizes camptothecin-mediated growth inhibition of Chinese hamster ovary cells by inhibition of DNA synthesis. Anticancer Drugs 10: 47–54. 10194547 10.1097/00001813-199901000-00007

[28] Ferte C, Massard C, Cohen A, Soria JC, Ederhy S (2010) Trastuzumab-induced cardiotoxicity: is it time for troponin for all patients? Am J Clin Oncol 35: 183–184. 10.1097/COC.0b013e318214e01f 22433996

[29] Hahn SM, Krishna CM, Samuni A, DeGraff W, Cuscela DO, et al (1994) Potential use of nitroxides in radiation oncology. Cancer Res 54: 2006s–2010s. 8137329

[30] Monti E, Cova D, Guido E, Morelli R, Oliva C (1996) Protective effect of the nitroxide tempol against the cardiotoxicity of adriamycin. Free Radic Biol Med 21: 463–470. 8886796 10.1016/0891-5849(96)00124-4

[31] Trnka J, Blaikie FH, Logan A, Smith RA, Murphy MP (2009) Antioxidant properties of MitoTEMPOL and its hydroxylamine. Free Radic Res 43: 4–12. 19058062 10.1080/10715760802582183 PMC2645131

[32] Simunek T, Sterba M, Popelova O, Adamcova M, Hrdina R, et al (2009) Anthracycline-induced cardiotoxicity: overview of studies examining the roles of oxidative stress and free cellular iron. Pharmacol Rep 61: 154–171. 19307704 10.1016/s1734-1140(09)70018-0

[33] Koyanagi N, Nagasu T, Fujita F, Watanabe T, Tsukahara K, et al (1994) In vivo tumor growth inhibition produced by a novel sulfonamide, E7010, against rodent and human tumors. Cancer Res 54: 1702–1706. 8137285

[34] Hamada J, Takeichi N, Kobayashi H (1988) Metastatic capacity and intercellular communication between normal cells and metastatic cell clones derived from a rat mammary carcinoma. Cancer Res 48: 5129–5132. 3409239

[35] Billingham ME, Mason JW, Bristow MR, Daniels JR (1978) Anthracycline cardiomyopathy monitored by morphologic changes. Cancer Treat Rep 62: 865–872. 667860

[36] Redon CE, Nakamura AJ, Sordet O, Dickey JS, Gouliaeva K, et al (2011) gamma-H2AX detection in peripheral blood lymphocytes, splenocytes, bone marrow, xenografts, and skin. Methods Mol Biol 682: 249–270. 21057933 10.1007/978-1-60327-409-8_18

[37] Levine RL, Wehr N, Williams JA, Stadtman ER, Shacter E (2000) Determination of carbonyl groups in oxidized proteins. Methods Mol Biol 99: 15–24. 10909073 10.1385/1-59259-054-3:15

[38] Aluise CD, Miriyala S, Noel T, Sultana R, Jungsuwadee P, et al (2011) 2-Mercaptoethane sulfonate prevents doxorubicin-induced plasma protein oxidation and TNF-alpha release: implications for the reactive oxygen species-mediated mechanisms of chemobrain. Free Radic Biol Med 50: 1630–1638. 21421044 10.1016/j.freeradbiomed.2011.03.009

[39] Shacter E, Weitzman SA (2002) Chronic inflammation and cancer. Oncology (Williston Park) 16: 217–226, 229; discussion 230–212. 11866137

[40] Redon CE, Dickey JS, Nakamura AJ, Kareva IG, Naf D, et al (2010) Tumors induce complex DNA damage in distant proliferative tissues in vivo. Proc Natl Acad Sci U S A 107: 17992–17997. 20855610 10.1073/pnas.1008260107 PMC2964229

[41] Doroshow JH, Synold TW, Somlo G, Akman SA, Gajewski E (2001) Oxidative DNA base modifications in peripheral blood mononuclear cells of patients treated with high-dose infusional doxorubicin. Blood 97: 2839–2845. 11313279 10.1182/blood.v97.9.2839

[42] Samuni Y, Cook JA, Choudhuri R, Degraff W, Sowers AL, et al (2010) Inhibition of adipogenesis by Tempol in 3T3-L1 cells. Free Radic Biol Med 49: 667–673. 20561604 10.1016/j.freeradbiomed.2010.05.028 PMC2904847

[43] Herman EH, Zhang J, Rifai N, Lipshultz SE, Hasinoff BB, et al (2001) The use of serum levels of cardiac troponin T to compare the protective activity of dexrazoxane against doxorubicin- and mitoxantrone-induced cardiotoxicity. Cancer Chemother Pharmacol 48: 297–304. 11710630 10.1007/s002800100348

[44] Bonner WM, Redon CE, Dickey JS, Nakamura AJ, Sedelnikova OA, et al (2008) gammaH2AX and cancer. Nat Rev Cancer 8: 957–967. 19005492 10.1038/nrc2523 PMC3094856

[45] Tanaka T, Halicka HD, Huang X, Traganos F, Darzynkiewicz Z (2006) Constitutive histone H2AX phosphorylation and ATM activation, the reporters of DNA damage by endogenous oxidants. Cell Cycle 5: 1940–1945. 16940754 10.4161/cc.5.17.3191 PMC3488278

[46] Nishio N, Oishi K, Machida K (2003) SST-2 tumor inoculation is a useful model for studying the anti-tumor immune response in SHR rats. Environ Health Prev Med 8: 1–5. 21432108 10.1007/BF02897936 PMC2723258

[47] Lu L, Wu W, Yan J, Li X, Yu H, et al (2009) Adriamycin-induced autophagic cardiomyocyte death plays a pathogenic role in a rat model of heart failure. Int J Cardiol 134: 82–90. 18619688 10.1016/j.ijcard.2008.01.043

[48] Arola OJ, Saraste A, Pulkki K, Kallajoki M, Parvinen M, et al (2000) Acute doxorubicin cardiotoxicity involves cardiomyocyte apoptosis. Cancer Res 60: 1789–1792. 10766158

[49] Mitchell JB, Anver MR, Sowers AL, Rosenberg PS, Figueroa M, et al (2012) The antioxidant tempol reduces carcinogenesis and enhances survival in mice when administered after nonlethal total body radiation. Cancer Res 72: 4846–4855. 22805306 10.1158/0008-5472.CAN-12-1879 PMC3445749

[50] Gurusamy N, Das DK (2009) Autophagy, redox signaling, and ventricular remodeling. Antioxid Redox Signal 11: 1975–1988. 19327038 10.1089/ars.2009.2524 PMC2848474

[51] Sishi BJ, Loos B, van Rooyen J, Engelbrecht AM (2013) Autophagy upregulation promotes survival and attenuates doxorubicin-induced cardiotoxicity. Biochem Pharmacol 85: 124–134. 23107818 10.1016/j.bcp.2012.10.005

[52] Mitchell JB, DeGraff W, Kaufman D, Krishna MC, Samuni A, et al (1991) Inhibition of oxygen-dependent radiation-induced damage by the nitroxide superoxide dismutase mimic, tempol. Arch Biochem Biophys 289: 62–70. 1654848 10.1016/0003-9861(91)90442-l

[53] Hahn SM, Tochner Z, Krishna CM, Glass J, Wilson L, et al (1992) Tempol, a stable free radical, is a novel murine radiation protector. Cancer Res 52: 1750–1753. 1551104

[54] Cunniff B, Benson K, Stumpff J, Newick K, Held P, et al (2013) Mitochondrial-targeted nitroxides disrupt mitochondrial architecture and inhibit expression of peroxiredoxin 3 and FOXM1 in malignant mesothelioma cells. J Cell Physiol 228: 835–45. 23018647 10.1002/jcp.24232 PMC3928986

[55] Roche M, Rondeau P, Singh NR, Tarnus E, Bourdon E (2008) The antioxidant properties of serum albumin. FEBS Lett 582: 1783–1787. 18474236 10.1016/j.febslet.2008.04.057

[56] Kuller LH, Eichner JE, Orchard TJ, Grandits GA, McCallum L, et al (1991) The relation between serum albumin levels and risk of coronary heart disease in the Multiple Risk Factor Intervention Trial. Am J Epidemiol 134: 1266–1277. 1755441 10.1093/oxfordjournals.aje.a116030

[57] Stevenson FT, Greene S, Kaysen GA (1998) Serum alpha 2-macroglobulin and alpha 1-inhibitor 3 concentrations are increased in hypoalbuminemia by post-transcriptional mechanisms. Kidney Int 53: 67–75. 9453001 10.1046/j.1523-1755.1998.00734.x

