# Supplementary figures and images for: Mito-Tempol and Dexrazoxane Exhibit Cardioprotective and Chemotherapeutic Effects through Specific Protein Oxidation and Autophagy in a Syngeneic Breast Tumor Preclinical Model
Source: PLoS One. 2013 Aug 5;8(8):e70575. doi: 10.1371/journal.pone.0070575 (PMC3734284; doi:10.1371/journal.pone.0070575)

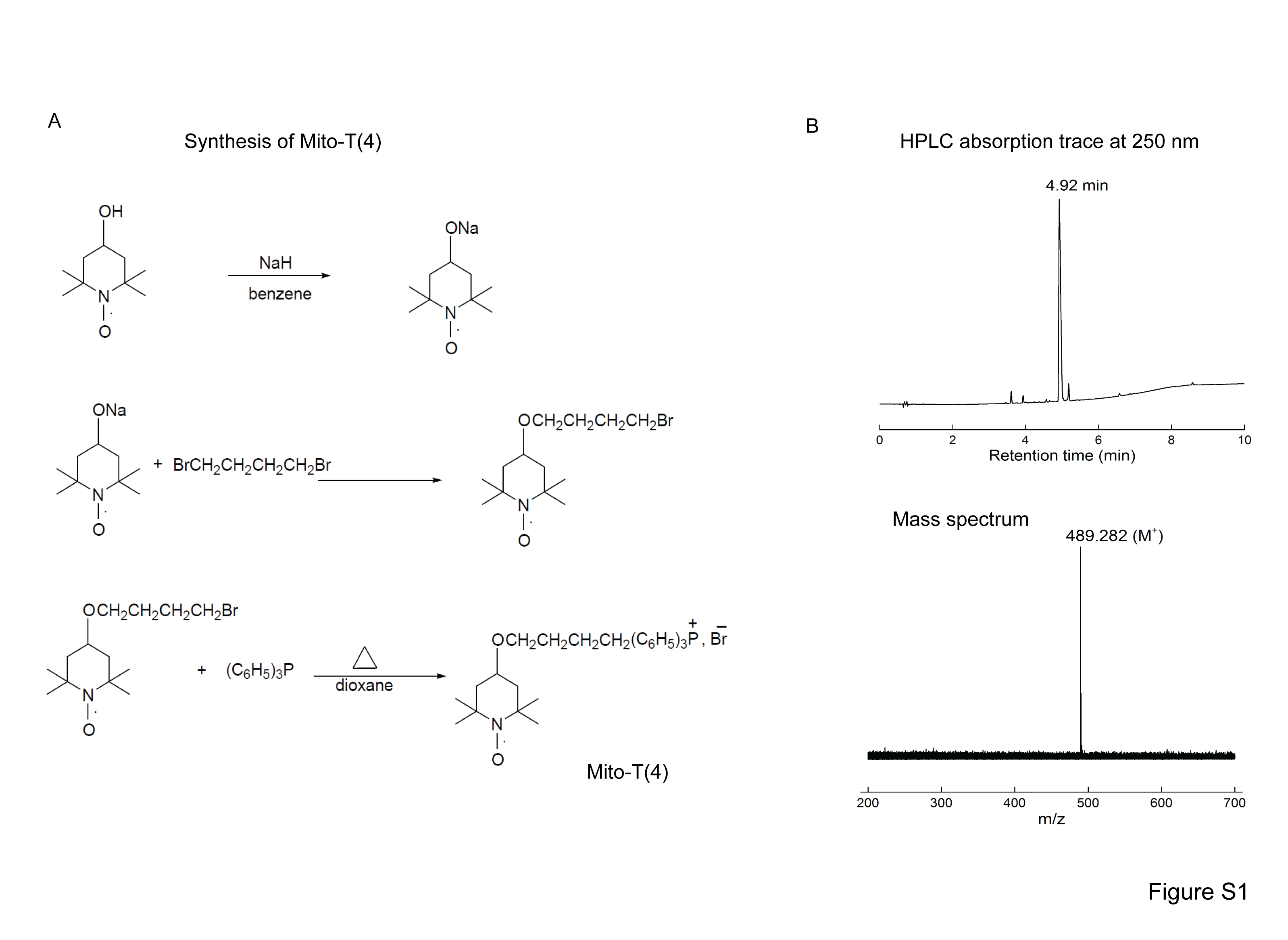

Supplement: Figure S1 — Scheme for synthesis of Mito-T (4). A, Mito-T (4) synthesis involved first the synthesis of Tempol-bromobutylether and then reacting this with triphenylphosphine to obtain Mito-T (4). B, Purity of the product was ascertained by performing HPLC and LC/MS (mass = 489). (TIF) [file pone.0070575.s001.tif]

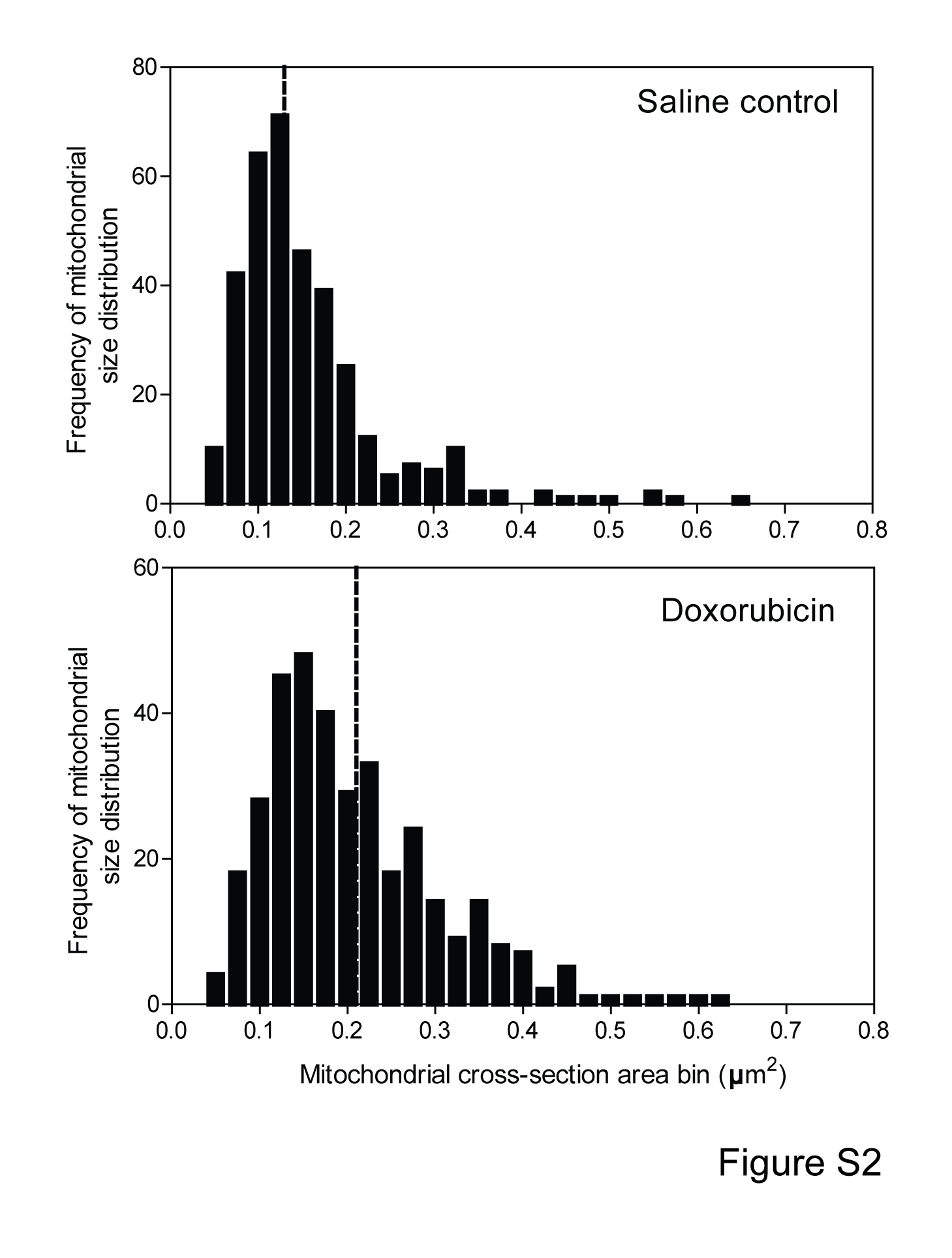

Supplement: Figure S2 — Comparison of mitochondrial cross-sections. The cross-sections showing mitochondrial swelling in Figure 3E were analyzed for quantitative differences in mitochondrial cross-section area. The area of 350 tumor cell mitochondria cross-sections from a representative animal tissue sample was determined from several random images of each sample (control and doxorubicin treated). The histograms shown indicate that most mitochondria cross-sections fall into the same range in both samples (from 0.1 to 0.2 μm2) but in doxorubicin treated tumor cells a significant amount of cross-sections also fall in a larger size range (from 0.2 to 0.4 μm2). The change in size distribution to larger size range of mitochondrial cross-sections is indicative of mitochondrial swelling. The median, mean, and standard deviation values were 0.14, 0.16+0.09 μm2 and 0.21, 0.23+0.11 μm2 for control and doxorubicin treated, respectively. The difference is statistically significant with a p<0.0001 using a one-tail, non-parametric Mann-Whitney test. The dotted lines on the graphs indicate the median value of the data set. (TIF) [file pone.0070575.s002.tif]

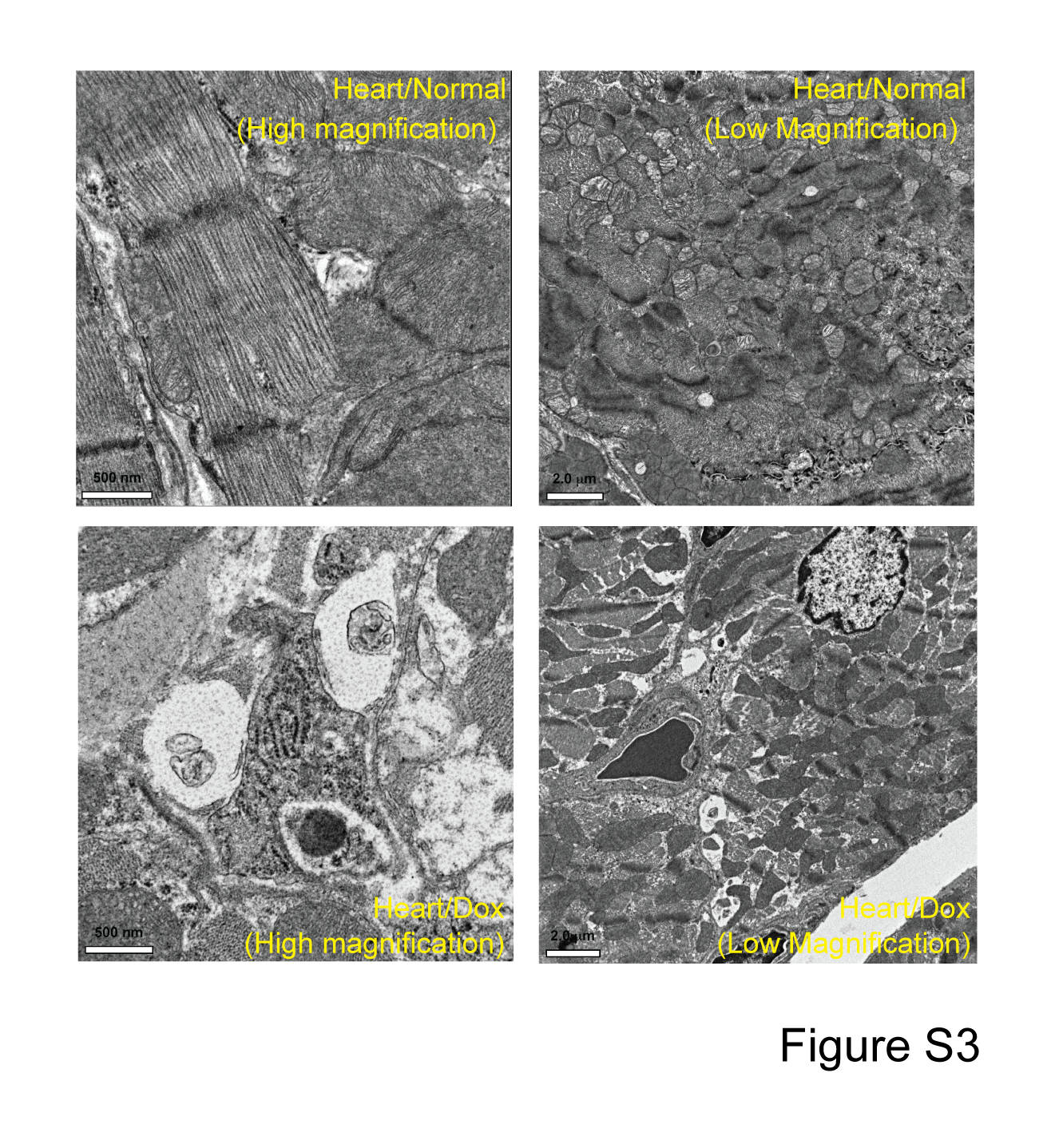

Supplement: Figure S3 — Ultrastructural analysis of autophagy in the cardiac tissue. Transmission electron microscopy was used to confirm autophagy in the heart tissue from SHR/SST-2 cells exposed to a cardiotoxic dose of doxorubicin. Low and high-magnification images are shown for clarity. Representative images of normal cells from saline treated tissue and damaged/autophagic cells from doxorubicin-treated tissue are shown. While autophagy was observed by TEM analysis in both saline and doxorubicin-treated heart tissue, a relative quantitative assessment could not be accurately made by TEM analysis due to the low occurrence of autophagic fraction of cells in the sarcomere matrix made up of myocytes and myofibrils. (TIF) [file pone.0070575.s003.tif]

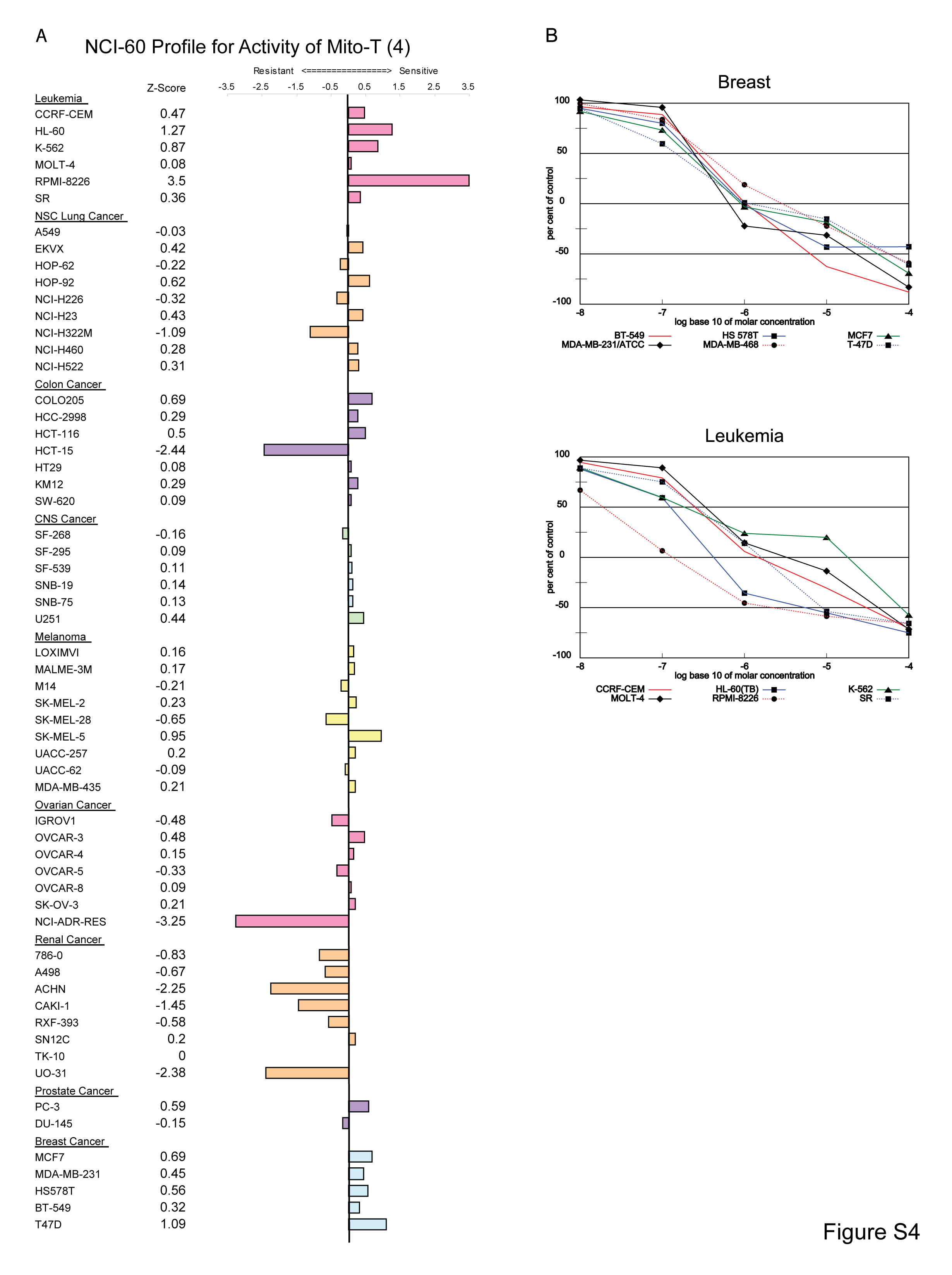

Supplement: Figure S4 — Anticancer activity of Mito-T (4) A, Graph illustrating the NCI-60 anticancer activity profile for Mito-T (4) as tested by the SRB assay in the NCI-60 anticancer drug screen cell line panel. Z-Score values for each cell line are plotted relative to the mean across all cell lines. Bars towards the right of the mean, such as the breast cancer and leukemia groups, are indicative of sensitivity relative to the mean across all cell lines. B, Dose response curves for Mito-T (4) in the breast cancer and leukemia groups of cell lines. Log base 10 of the molar concentrations used in the SRB assay are plotted against the percentage growth in cells over 72 h. (TIF) [file pone.0070575.s004.tif]

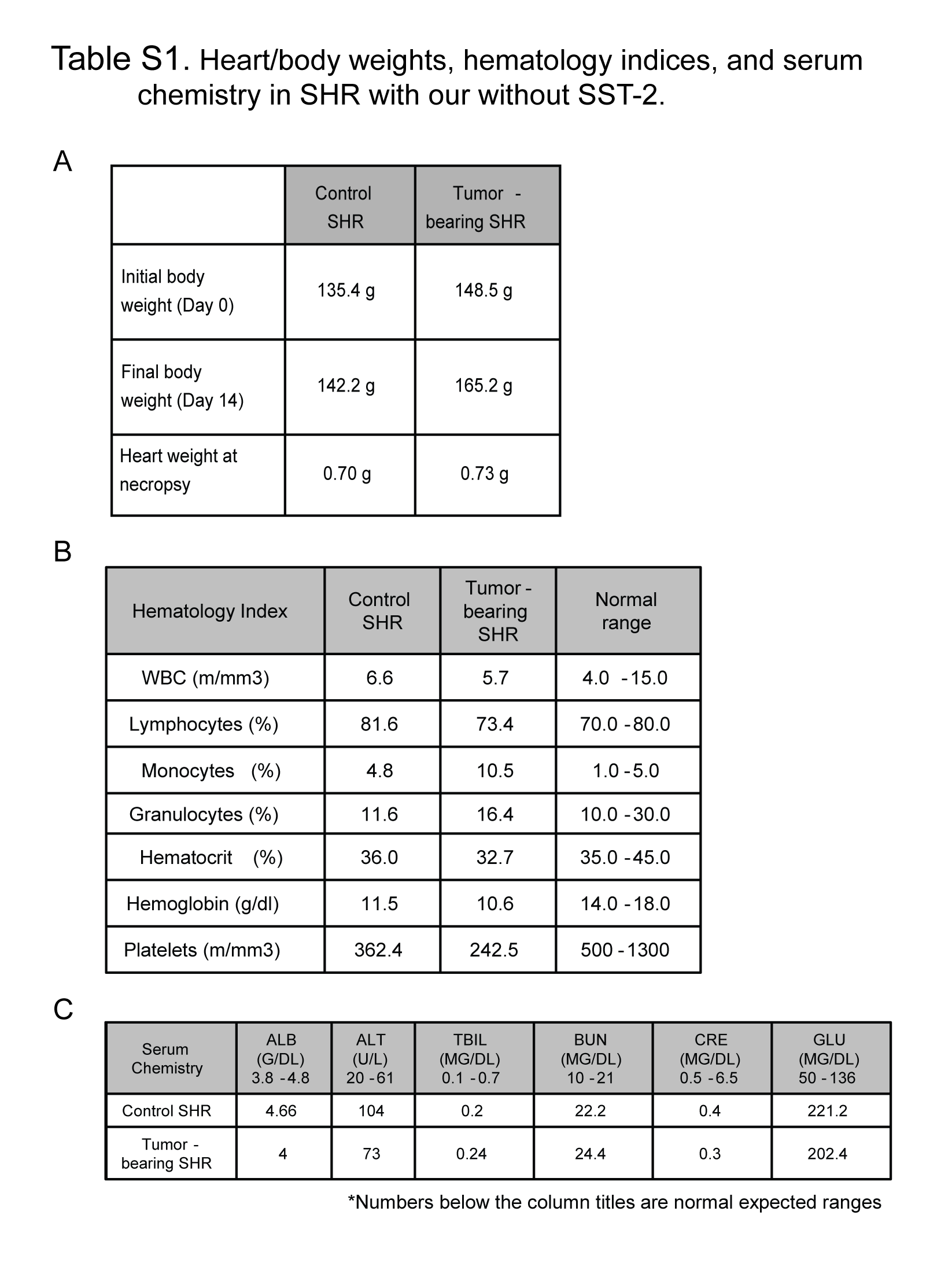

Supplement: Table S1 — Heart/body weights, hematology indices, and serum chemistry in SHR with or without SST-2 cells. A, Control and tumor-bearing SHR were weighed before and 14 days after SST-2 implantation. The hearts were weighed post-necropsy on day 14. B, Whole blood from non-tumor bearing and tumor-bearing SHR was analyzed for white blood cell (WBC), lymphocyte, monocyte, granulocyte, hematocrit, hemoglobin, and platelet numbers. These data were compared to the expected values in SHR animals shown in the far right column. C, Serum from non-tumor bearing (Control SHR) and tumor-bearing SHRs was analyzed post-necropsy to assess the impact of tumor growth on animal health. The table shows the mean serum concentrations of albumin (ALB), alanine aminotransferase (ALT), total bilirubin (TBIL), blood urea nitrogen (BUN), creatinine (CRE), and glucose (GLU). (TIF) [file pone.0070575.s005.tif]

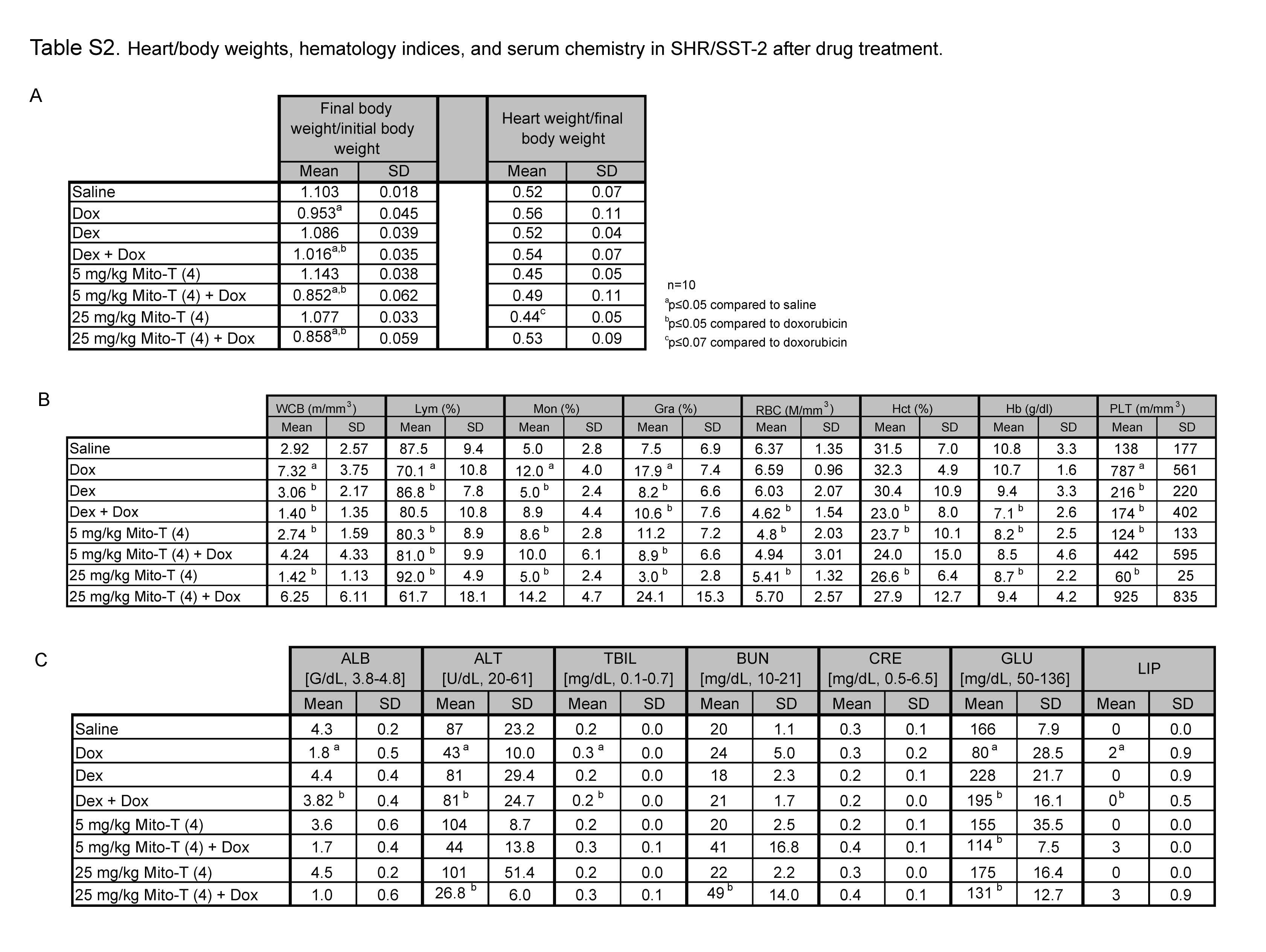

Supplement: Table S2 — Heart/body weights, hematology indices, and serum chemistry in SHR with SST-2 cells after drug treatment. The analysis of weights, hematology and serum chemistry were performed similar to Table S1. The animals were treated as indicated in the first column of each set of data. A, Ratios of final to initial body weight and heart to body weight. B, Whole blood analyses. C, Serum chemistry. (TIF) [file pone.0070575.s006.tif]
